# Genetic Surfaceome *E. coli* Reprogramming Enables Selective Water Oxidation

**DOI:** 10.1002/adma.202508100

**Published:** 2025-08-15

**Authors:** Graziela C. Sedenho, Jéssica C. Pacheco, Melanie Gut, Filipe C. D. A. Lima, Sunanda Dey, Frank N. Crespilho, Ariel L. Furst

**Affiliations:** ^1^ São Carlos Institute of Chemistry University of São Paulo (USP) São Carlos São Paulo 13566–590 Brazil; ^2^ Department of Chemical Engineering Massachusetts Institute of Technology Cambridge MA 02139 USA; ^3^ Federal Institute of Education, Science, and Technology of São Paulo Matão São Paulo 15991‐502 Brazil

**Keywords:** bilirubin oxidase, biomaterials, electrocatalysis, water oxidation

## Abstract

Programming catalytic behavior at the microbial genome level is a frontier in synthetic biology with direct impact on bioelectrocatalysis. A key challenge is the coordinated control of gene expression, localization, folding, and cofactor maturation required to achieve proper bioelectrocatalytic activity. Here, a synthetic operon in *Escherichia coli* is engineered to reprogram its surfaceome for selective water oxidation. Using orthogonal IPTG‐inducible control and codon‐optimized expression, a fungal bilirubin oxidase (BOD) displayed at the cell surface is produced by ice nucleation protein anchoring (BOD‐*E. coli*). Post‐overexpression copper catalytic site reconstitution provides an active holoenzyme. The developed engineered living material performs water oxidation at near‐zero overpotential (27 mV at pH 9.1), with complete suppression of the oxygen reduction reaction. These results show how regenerable microbial platforms can be designed for selective catalysis and artificial photosynthesis.

## Graphical abstract

A novel enzymatic material based on bilirubin oxidase (BOD) surface‐expressed on *E. coli* for effective electrocatalysis is reported. With this material, highly efficient and selective water oxidation is achieved, a critical half reaction for synthetic photosynthesis.

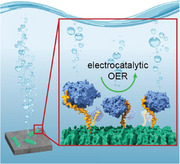

## 1 Introduction

Artificial photosynthesis through biological systems remains one of the most ambitious frontiers in synthetic biology and metabolic engineering. Central to this challenge is the oxygen evolution half‐reaction, which, in natural photosynthesis, is catalyzed by the oxygen‐evolving complex, a Mn_4_CaO_5_ cluster embedded within Photosystem II. The structural and redox complexity of this multinuclear cofactor has rendered its reconstruction outside of the thylakoid membrane nearly impossible. As a result, bioinspired efforts have focused on developing alternative water oxidation systems. However, these approaches often rely on purified redox enzymes or synthetic organometallic catalysts, both of which present challenges in terms of stability, cost, and scalability.

An emerging alternative is to leverage cell‐surface enzyme display to develop engineered living materials (ELMs) as catalytic systems. These materials offer a novel avenue to repurpose engineered biological systems for innovative and scalable applications, including biosensing, green energy production, and sustainable synthesis of value‐added compounds.^[^ ^1^, ^2^, ^3^ ^]^ By genetically fusing target proteins to membrane‐anchoring motifs, they can be localized to the exterior of living cells in an active conformation. Using microbial platforms, such as *Escherichia coli* (*E. coli*), eliminates the need for enzyme purification and supports self‐regenerating biocatalysts. Redox‐active enzymes displayed on the cell surfaces exhibit longer‐term operational stability and higher catalytic performance when compared to the purified enzymes.^[^ ^4^, ^5^, ^6^ ^]^ This stability has been attributed to the presence of microbial cell surface and the close packing of the proteins on the cell as stabilizing elements, as this local environment better mimics the native environment of the enzyme.^[^ ^7^, ^8^ ^]^ In addition, this approach enables biocatalyst regeneration, as enzymes are constantly recycled in native cell metabolism, allowing new enzymes to be constantly expressed. This advantage enables longer reaction run times and reduces biocatalyst production costs. Enzyme isolation and purification represent as much as 80% of the total production cost of proteins. Therefore, surface expression circumvents the costly, laborious, and time‐consuming enzyme purification procedures.^[^ ^4^, ^5^, ^6^ ^]^

Among redox‐active enzymes, multicopper oxidases (MCOs), including laccase and bilirubin oxidase (BOD), are particularly interesting for surfaceome engineering. These enzymes coordinate copper ions at distinct T1, T2, and T3 centers to catalyze multi‐electron redox reactions, most commonly the four‐electron reduction of molecular oxygen.^[^ ^9^, ^10^, ^11^ ^]^ However, under specific electrochemical conditions, recent studies have reported that MCOs also catalyze the reverse reaction: the water oxidation reaction (WOR).^[^ ^12^, ^13^, ^14^, ^15^ ^]^ This functional reversal is hypothesized to result from microenvironmental engineering near the active site, where proton gradients, hydration dynamics, and local redox potential shift the equilibrium and promote O─O bond formation via high‐valent Cu(III)‐oxo intermediates.^[^ ^15^ ^]^

Despite their promise, surface expression of MCOs presents significant challenges. The extracytoplasmic localization of these enzymes removes them from native metallochaperones and folding assistants, often resulting in incomplete metalation and inactive apoenzymes. Strategies such as in vitro copper reconstitution, co‐expression of copper‐trafficking proteins, or controlled Cu^2^⁺ supplementation have shown partial success,^[^ ^16^ ^]^ but full site‐specific maturation of surface‐displayed MCOs has remained a key limitation. Moreover, achieving selective WOR over oxygen reduction—an uncommon phenotype in this enzyme class—requires precise genetic control over both enzyme localization and interface tuning, as well as thermodynamic steering via hydration shell modulation and proton‐coupled electron transfer (PCET) pathways. To address these interconnected challenges, we present a genome‐level chassis reprogramming strategy in *E. coli*, combining synthetic operon architecture, orthogonal isopropyl β‐D‐1‐thiogalactopyranoside (IPTG)‐inducible control, and membrane‐targeted expression of a codon‐optimized fungal BOD. Using a fusion anchor, we achieved stable outer membrane display of BOD in its apo‐form, followed by in situ Cu^2^⁺ metalation to yield a fully functional holoenzyme.

## 2 Results and Discussion

### 2.1 Genetic Reprogramming of the *E. coli* Surfaceome

Though several surface expression tags have been developed for use in *E. coli*, most are cytotoxic and limit either the viability of the cells produced or the amount of protein on the cell surface. We have previously employed the ice nucleation protein (INP) from *Pseudomonas syringae* with the NC‐terminal fusion (INPNC: fusion of the *N*‐terminal membrane domain INP_N_ and the *C*‐terminal extracellular domain INP_C_) for the surface display of heterologous proteins.^[^ ^6^, ^17^, ^18^, ^19^ ^]^ The INP is an outer‐membrane protein with a hydrophobic *N*‐terminal domain that links the protein to the membrane by glycosylphosphatidylinositol anchoring, while the remainder of the full‐length protein is hydrophilic and specifically interacts with water molecules to organize them in such a way that the barrier to ice formation is lowered.^[^ ^20^, ^21^, ^22^, ^23^ ^]^ Because of the prevalence of this class of proteins and their importance in geochemical processes, they have been well‐characterized, and their interactions with water molecules well‐established. Unlike many other peptide tags evaluated for the surface display of proteins on *E. coli*, we do not see cytotoxicity with the INP sequence employed.

**Figure 1:**
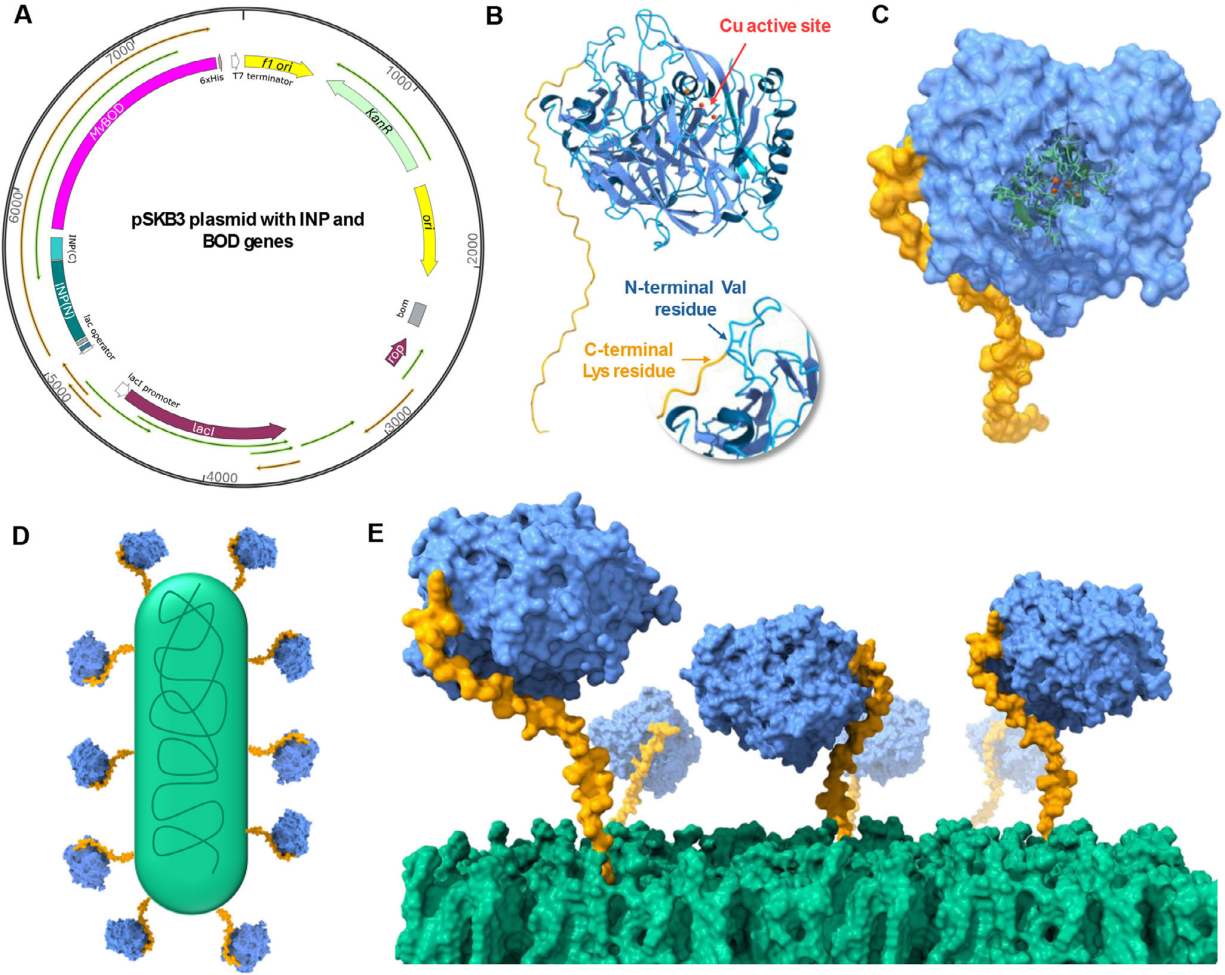
Synthetic Gene Construct and Structural Model of Surface‐Anchored BOD via INP Fusion in *E. coli*. A) Designed a plasmid with BOD from *Myrothecium verrucaria* and INP genes. B) Structure of BOD‐fused INP. Zoomed view highlights the Val 1 *N*‐terminal residue of BOD, where the *C*‐terminal extracellular domain (INPC) of INP protein is bound. C) Molecular surface model of BOD‐fused INP highlighting the BOD Cu‐active site. D) Scheme representing BOD anchored to *E. coli* surface by the INP protein and its (E) zoomed view. The INP extracellular domain, BOD, and lipid bilayer of the cell surface are shown in yellow, blue, and green, respectively.

Prior to plasmid construction (**Figure** **1**A), a molecular model of INP‐BOD was generated to provide confidence that the *N*‐terminal fusion would not interfere with the BOD folding. The structure of BOD in combination with the extracellular domain of INP was obtained from the AlphaFold Protein Structure Database^[^ ^24^, ^25^ ^]^ ID AF‐Q12737‐F1‐v4 with a mean pLDDT (predicted Local Distance Difference Test) score of 94.66, indicating high confidence in the majority of the proteins. The model is shown in Figure 1B–E, both in the absence and presence of the membrane, providing a detailed representation of the protein‐membrane environment. Based on the structural prediction of this construct, the INP maintains a random secondary structure facing the BOD surface even when anchored in the membrane (BOD‐*E. coli*, Figure 1D,E). This tail is distal to the Cu‐active site in the enzyme, minimizing the risk of the INP tail interfering with catalysis.

Based on this protein model, a plasmid for surface expression of fungal BOD was constructed using a pSKB3 vector. The 1602‐base pair (bp) BOD‐coding gene was designed from the 543‐residue protein sequence of BOD from *Myrothecium verrucaria* and codon optimized for *E. coli* expression. The INP tag was inserted at the *N*‐terminus of the BOD sequence, and the complete plasmid (pSKB3‐INP‐BOD, Figure 1A) was generated through Gibson assembly. The design of plasmid, primers, and genes, as well as polymerase chain reaction (PCR) amplification, are detailed in Figures S1–S3 and Table S1 (Supporting Information). The pSKB3‐INP‐BOD plasmid was sequenced to confirm that no mutagenesis occurred and transformed into BL21 *E. coli* for expression (Figures S4 and S5, Supporting Information). IPTG‐induced expression was optimized, with overexpression confirmed by gel electrophoresis (Figure S6, Supporting Information). Based on prior optimization of this surface expression system, INP‐BOD expression is expected to yield between 50 000 and 70 000 proteins per cell using optimized induction conditions.^[^ ^17^, ^18^, ^19^ ^]^

### 2.2 Cu^2^⁺ Reconstitution Drives Surface Activation of apoBOD

**Figure 2:**
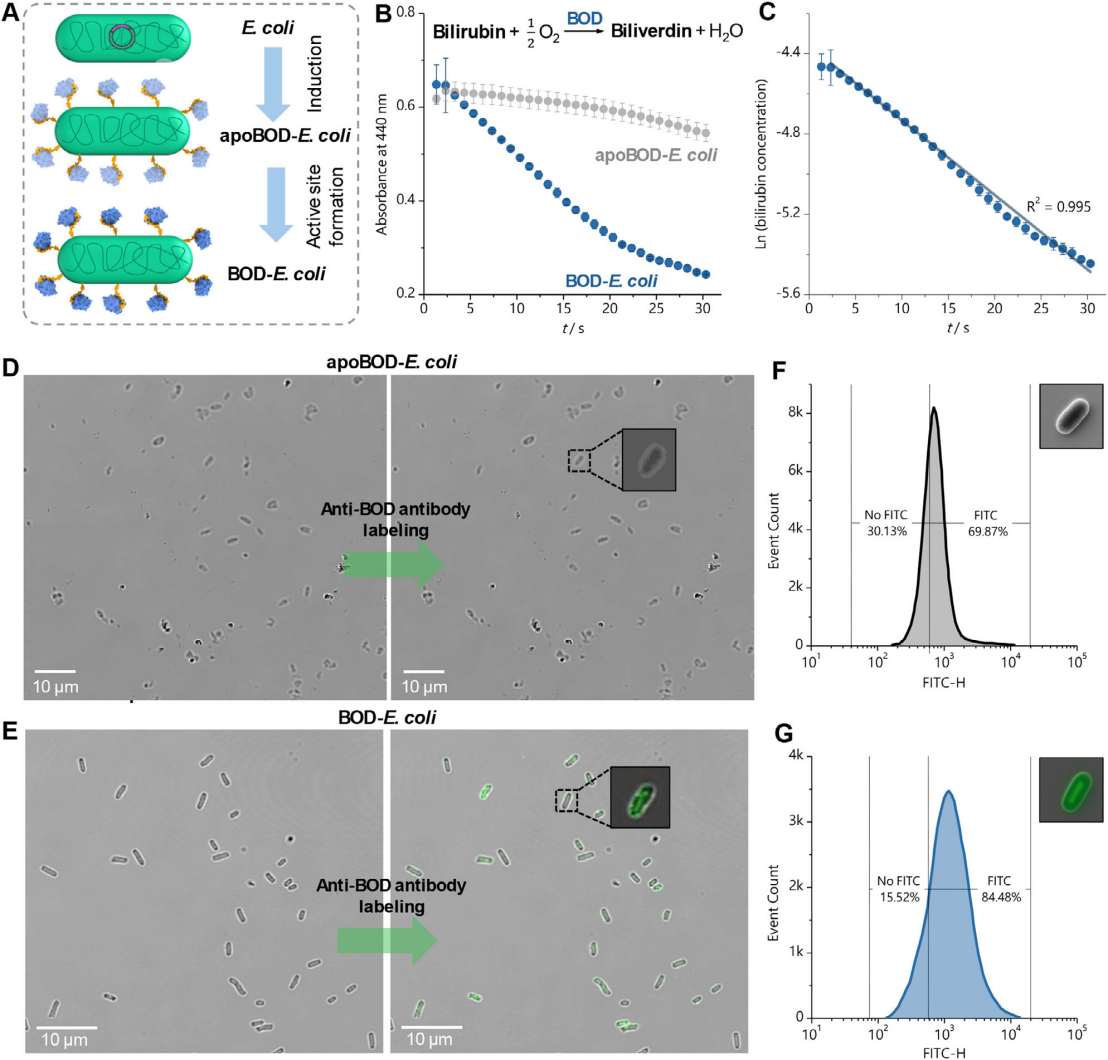
Expression, Reconstitution, and Characterization of BOD displayed on *E. coli* surface. A) Scheme representing the obtention of active BOD‐displayed *E. coli* cell surface. B) Monitoring of bilirubin consumption by absorbance at 440 nm and 37 °C over time, in 0.2 mol L^−1^ Tris‐HCl (pH 8.4) initially containing 0.04 mmol L^−1^ bilirubin, as substrate, in the presence of BOD‐*E. coli* (OD_600_ = 0.1, blue dots) and apoBOD‐*E. coli* (OD_600_ = 0.1, gray dots). C) Linear correlation of substrate concentration versus natural logarithm of time. Confocal microscopy of (D) apoBOD‐*E. coli* and (E) BOD‐*E. coli* labeled with fluorescent anti‐BOD antibody, images of phase contrast (left) and merged with the FTIC channel (right). Insets: zoomed views of the labeled cells. E) Histograms of the flow cytometric analysis indicating the FITC and non‐fluorescent populations of apoBOD‐*E. coli* and BOD *E. coli*. Insets: confocal microscopy images from the insets of panels D and E after digital processing using Python 3.10 with the libraries OpenCV, Pillow (PIL), and NumPy to enhance signal clarity.

MCOs, including BOD, contain four Cu^2+^ ions in their active sites, classified in Cu Type 1 (one ion), Type 2 (one ion), and Type 3 (two ions). These ions are crucial for both catalytic conversions and electron transfer reactions.^[^ ^15^, ^26^, ^27^ ^]^ With our construct, the BOD was expressed on the surface of the *E. coli* in its apo form (without Cu^2+^ chelated in its active sites). The reconstitution of this Cu‐based active site to generate the active, holo‐enzyme is summarized in **Figure** **2**A and was optimized as described in Figures S7 and S8 and Tables S2–S4 (Supporting Information). ICP‐MS analysis enabled confirmation of Cu^2+^ complexation to the cell surface and was used to approximate the average number of Cu^2+^ ions per BOD protein to be ~2 (Tables S5–S7, Supporting Information). No copper could be detected in control samples (Tables S5 and S6, Supporting Information). The ICP‐MS results indicate that 50% of BOD molecules on the *E. coli* surface were effectively reconstituted, as each BOD molecule is expected to have four Cu^2+^ ions. Based on that, it is estimated that there are 25 000–35 000 holo‐BOD molecules per cell. The successful generation of the holo‐enzyme was confirmed via activity assay monitoring the consumption of the native BOD substrate, bilirubin. BOD oxidizes this substrate to biliverdin in the presence of molecular oxygen, which can be monitored as an absorbance decrease at 440 nm over incubation time as the bilirubin is oxidized (Figure 2B). The apoBOD‐*E. coli* shows little activity toward bilirubin oxidation, while the successfully reconstituted BOD‐*E. coli* rapidly oxidizes nearly all of the bilirubin present within half an hour. From this simple assay, we confirmed that the Cu^2+^ reconstitution was successful and that our *E. coli* cells were now decorated with active metalloenzyme. In addition, the linear dependence of the bilirubin concentration with the natural logarithm of time (Figure 2C) indicates a first‐order biocatalytic reaction to this substrate under the employed experimental conditions. First‐order enzymatic reactions indicate that the substrate concentration is very low (bilirubin concentration << Michaelis–Menten constant) and the reaction rate is proportional to this substrate concentration.

For the application of this ELM in bioelectrocatalytic systems, the BOD must be expressed on the surface of the cells to ensure that the enzyme is in direct contact with the electrode surface for direct electron transfer from (or to) the metal active site. Internal expression of the protein would necessitate electron transfer across the insulating cell membrane, which often requires small‐molecule mediators that slow the reaction due to diffusion limitations. Surface expression was also expected to promote substrate conversion by negating the need for substrate diffusion or transport into the cell. To investigate the surface expression of BOD, in situ protein characterization by confocal microscopy and flow cytometry was performed (Figure 2D–G; Figure S12, Supporting Information). Cells expressing BOD were labeled with either an anti‐BOD antibody or with an anti‐His_6_‐antibody, as the BOD contained a *C*‐terminal His_6_ tag. These complementary labeling approaches were anticipated to enable the visualization of BOD localized to the cell surface. Additionally, microbes were labeled with 4′,6‐diamidino‐2‐phenylindole (DAPI) fluorescent dye for cell staining (Figures S10 and S11, Supporting Information). The fluorescence signal (Figure 2E, green) from cells labeled with anti‐BOD antibody indicates the presence of BOD on the cell surface for samples with holo protein, as expected. This trend was also observed using anti‐His_6_ antibodies, indicating that BOD is expressed on the *E. coli* cell surface (Figure S12, Supporting Information). Interestingly, no fluorescent signal is observed with the apoBOD‐*E. coli* sample (Figure 2D), which is consistent with improved antibody binding upon proper protein folding^[^ ^28^ ^]^ when the Cu^2+^ active sites are reconstituted as compared to the apoenzyme. Further, results from flow cytometry show similar results, with higher levels of overall fluorescence for the holoprotein (Figure 2G) as compared to the apoprotein (Figure 2F). Taken together, these results confirm that the protein is expressed on the surface of the *E. coli* and further validate that the holoenzyme is folded as the native protein following reconstitution, as this is the only protein form recognized by the anti‐BOD antibody.

### 2.3 Selective Water Oxidation

**Figure 3:**
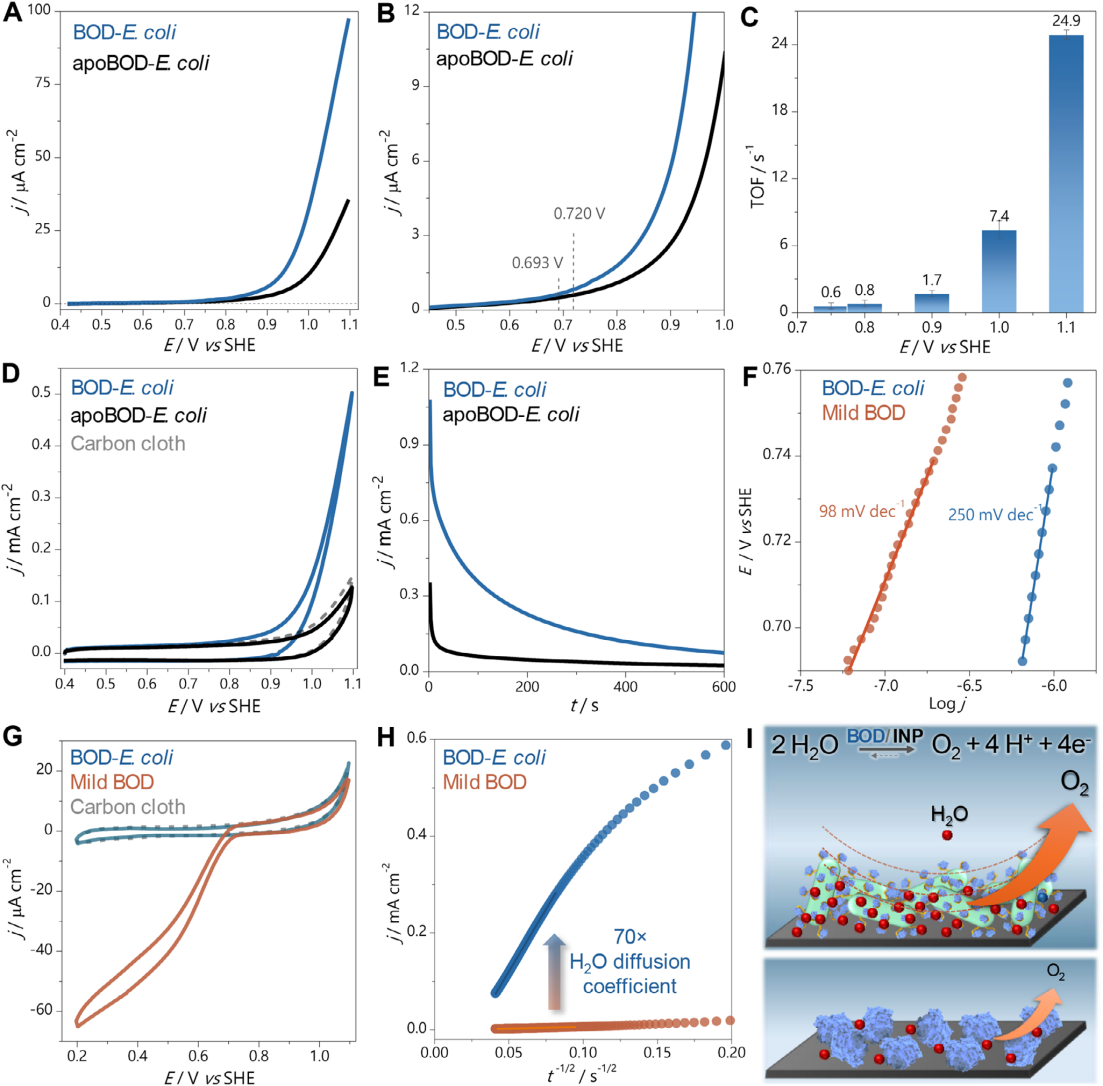
Bioelectrocatalytic behavior of BOD displayed on the *E. coli* surface. A) Linear voltammograms at 1 mV s^−1^ recorded with BOD‐*E. coli* and apoBOD‐*E. coli* immobilized on a carbon cloth electrode in 0.1 mol L^−1^ phosphate solution (pH 9.1). B) Zoomed view of A, highlighting the thermodynamic and experimental onset potential values for WOR. C) Estimated TOF values according to the applied potential. D) Cyclic voltammograms at 25 mV s^−1^ recorded with BOD‐*E. coli* and apoBOD‐*E. coli* immobilized on a carbon cloth electrode and a bare carbon cloth electrode. E) Current–time curves recorded at 1.10 V with BOD‐*E. coli* and apoBOD‐*E. coli* immobilized on a carbon cloth electrode in 0.1 mol L^−1^ phosphate solution (pH 9.1). F) Potential versus log *j* curve (Tafel plot) of WOR for BOD‐*E. coli* and purified mild BOD‐modified electrodes. G) Cyclic voltammograms at 25 mV s^−1^ recorded with BOD‐*E. coli* and purified mild BOD immobilized on carbon cloth and bare carbon cloth electrodes in 0.1 mol L^−^^1^ phosphate buffer solution (pH 7.2). H) Correlation of *j* with *t* ^−1/2^ for BOD‐*E. coli* and mild BOD‐modified electrodes at 1.10 V, indicating enhanced O_2_ diffusion coefficient for BOD‐*E. coli* modified electrode. All measurements were performed at room temperature and in air‐saturated electrolytes. I) Representations of the BOD anchored by INP on the *E. coli* surface (top) and purified mild BOD (bottom) immobilized on the carbon electrode. Water diffusion can lead to an equilibrium shift favoring WOR over ORR with BOD‐*E. coli*.

Following confirmation that the BOD was expressed on the surface of cells and was properly folded and active following Cu^2+^ reconstitution, the electrocatalytic behavior of the BOD‐*E. coli* was then evaluated. Initial electrochemical conditions were determined based on those previously employed for BOD‐catalyzed WOR.^[^ ^15^ ^]^ BOD‐*E. coli* were physically adsorbed on carbon cloth, which provides a high surface area for interaction between the electrode and the proteins on the cells while also serving as a current collector. A sample of 50 µL of BOD‐*E. coli* (OD_600_ = 2.2) was dropped‐casted on a 1 cm^2^ carbon cloth electrode. This volume resulted in an estimated holo‐BOD loading of ≈0.35 µg cm^−2^ (see calculation in the Supporting Information). Quasi‐steady‐state voltametric curves (**Figure** **3**A,B) indicate that the onset potential for the WOR is 0.720 ± 0.010 V at pH 9.1, corresponding to an exceptionally small overpotential of 27 mV (thermodynamic potential equals 0.693 V vs standand hydrogen electrode, SHE, pH 9.1) when the carbon electrode is modified with active BOD‐*E. coli*. This value is similar to those previously reported for commercially‐available BOD under similar conditions, and much smaller than inorganic electrocatalysts (Table S8, Supporting Information).^[^ ^15^ ^]^ Holo‐BOD catalysis for WOR reached an estimated turnover frequency (TOF) of 49.8 ± 0.8 s^−1^ at 1.10 V (0.410 V‐overpotential, Figure 3C). This TOF value is higher than those reported for several non‐noble metal‐based electrocatalysts (Table S8, Supporting Information), indicating high catalytic activity of BOD‐*E*. coli toward WOR. In contrast, no bioelectrocatalysis is observed on bare carbon cloth or when the apoBOD‐*E. coli* is used for carbon cloth modification (Figure 3D). These results confirm that the electrocatalytic WOR behavior is the result of the activity of the fully reconstituted BOD on the *E. coli*. At 25 mV s^−1^ (Figure 3D), oxidative faradaic currents are observed at potentials (*E*) higher than 0.80 V, reaching a current density (*j*) of 0.50 mA cm^−2^ at 1.10 V (1.44 A mg^−1^ holo‐BOD, Figure S13, Supporting Information). Unlike apoBOD‐*E. coli* and bare carbon cloth electrodes reach current densities of only 0.13 and 0.15 mA cm^−2^, respectively, at the same potential (0.410 V‐overpotential).

Additional electrochemical characterization further confirmed these observations. Current–time measurements at a constant potential are consistent with the results obtained by cyclic voltammetry. At 1.10 V (Figure 3E), the apoBOD‐*E. coli*‐modified electrode shows minimal oxidative current (24 µA cm^−2^), while the active BOD‐*E. coli* reaches a steady‐state current of 75 µA cm^−2^ (0.21 A mg^−1^ holo‐BOD, Figure S13, Supporting Information). The anodic charge over the 600 s was calculated to be 0.13 and 0.03 mC with active BOD‐*E. coli* and apoBOD‐*E. coli* (control condition), respectively. These levels of charge generation indicate an overall faradaic efficiency (FE) of ≈77% for O_2_ production by BOD‐*E. coli*, which represents 0.27 µmol O_2_ produced over 600 s at 1.10 V. In addition, BOD‐*E. coli* provides higher faradaic current densities toward WOR compared to our previous findings using purified mild BOD adsorbed on carbon‐based electrodes (22 µA cm^−2^), under the same experimental conditions.^[^ ^15^ ^]^ Taken together, these results confirm that the holoprotein is responsible for WOR with high FE and that the surface‐expressed protein is significantly more efficient than the isolated mild BOD. Long‐term current‐time curve at 1.10 V performed with BOD‐*E. coli*, and compared with isolated mild BOD and uninduced *E. coli* cells, evidence shows the stable and efficient WOR promoted by BOD‐*E. coli* (Figure S9, Supporting Information).

To further investigate the electrocatalytic properties of BOD‐*E. coli* as it compares to the purified mild BOD, Tafel plots were analyzed (Figure 3F). The Tafel slope is a well‐established metric for assessing rates and mechanisms of electrocatalytic reactions.^[^ ^29^ ^]^ We calculated Tafel slopes for quantitative comparison of electrodes modified with BOD‐*E. coli* and purified BOD from *Myrothecium verrucaria*. Tafel plots (Figure 3F) were constructed based on quasi‐steady‐state voltammetric curves (Figure 3A; Figure S10, Supporting Information), with Tafel slopes extrapolated from fitting the low overpotential linear regions. These regions are those where kinetics are governed by charge transfer without mass transport limitations (see details in the Supporting Information).^[^ ^30^ ^]^ Lower Tafel slope values indicate faster kinetics, as smaller overpotentials are required to reach higher current densities. The Tafel slope for the BOD‐*E. coli* electrode was estimated to be 250 ± 2 mV dec^−1^, comparable to inorganic Cu‐based electrocatalysts operating in strongly alkaline electrolytes (see Table S8, Supporting Information). In contrast, the purified mild BOD‐modified electrode showed a Tafel slope of 98 ± 3 mV dec^−1^. The lower slope suggests that the purified protein transfers electrons to the electrode faster than the BOD‐*E. coli* under WOR conditions. This behavior is expected, as the active sites of the free enzymes are likely closer to the electrode and more favorably oriented for charge exchange than those attached to the *E. coli* surface. This limitation of surface‐displayed enzymes is not significant and is expected to be readily mitigated through the incorporation of nanostructured carbon materials such as nanoparticles. These materials have been reported to improve contact with enzyme active sites for more efficient direct electron transfer.^[^ ^10^ ^]^

Interestingly, unlike purified mild BOD from *Myrothecium verrucaria* under similar conditions,^[^ ^15^ ^]^ heterologous BOD‐*E. coli* does not show reversible O_2_/H_2_O bioelectrocatalytic behavior at pH 9.1, as no faradaic currents for oxygen reduction reaction (ORR) are observed in the voltametric curves (Figure 3A,D). Even in near‐neutral electrolyte (pH 7.2), where the standard activity of reducing O_2_ (thermodynamic potential equals 0.805 V vs SHE, pH 7.2) is favored over WOR by BOD, no electrochemical evidence of ORR is observed with BOD‐*E. coli* (Figure 3G). The results support the selectivity of the BOD displayed on *E. coli* for WOR electrocatalysis over ORR, which is hypothesized to be due to a shift in the thermodynamic equilibrium of the reaction in favor of WOR. A thermodynamic equilibrium for a reversible reaction can be shifted by significantly increasing reactant (substrate) concentrations.^[^ ^31^, ^32^ ^]^ We hypothesize that the surface‐expressed BOD experiences a higher local concentration of water (the substrate for WOR) as compared to the purified protein. This effect can be achieved by enhanced substrate mass transfer from the bulk electrolyte to the BOD‐*E. coli*‐modified electrode compared to the purified mild BOD‐modified electrode. To investigate this hypothesis, the diffusion of water to the electrode surface was analyzed based on current–time curves at a constant potential, applying the Cottrell equation (see details in the Supporting Information). The calculations indicate that the apparent diffusion coefficient is more than 70 times higher when using BOD‐*E. coli* compared to purified mild BOD (Figure 3H). As previously discussed, the high hydrophilicity of the INP extracellular domain and the repeating domain containing an alternating serine‐threonine segment is the basis of the BOD‐*E. coli* selectivity toward WOR. The complete ORR suppression is also related to this feature, as the non‐polar O_2_ molecules (the substrate for ORR) are repelled by the hydrophilic and polar residues of the INP extracellular domain. The exclusion of O_2_ in the BOD microenvironment leads to a complete equilibrium shift for the WOR.

The increase in substrate diffusion coefficient for the surface‐expressed protein as compared to the isolated protein supports our initial hypothesis that mass transport to the enzyme is enhanced in the whole‐cell system. We then further hypothesized that this difference is due to the presence of the INP sequence on the surface of the cell. It is well‐established that INPs induce the formation of ice crystals in native systems by interacting with surrounding water molecules to control their local organization.^[^ ^33^, ^34^, ^35^ ^]^ The *C*‐terminal extracellular domain of INP is highly hydrophilic, and a repeating domain containing a unique alternating hydrophobic‐hydrophilic segment (serine‐threonine) specifically interacts with water molecules (Figure S14, Supporting Information) to organize them and decrease the kinetic barrier for ice formation.^[^ ^34^, ^35^ ^]^ Because the behavior of this amino acid sequence in an aqueous environment is known to impact the energy landscape of local water molecules, it is reasonable to associate these interactions with improved mass transport of bulk water to the BOD active site when the INP tag is incorporated. Therefore, we hypothesize that the INP extracellular domain interacts with surrounding water layers, providing enhanced substrate (water) mass transfer to the BOD catalytic site (Figure 3I).^[^ ^31^, ^32^ ^]^

### 2.4 Genetic Control of Interface‐Encoded Catalysis in Living Systems

The genetic architecture underlying our system is composed of a synthetic operon inserted into the high‐copy plasmid pSKB3, featuring a codon‐optimized open reading frame (ORF) for the fungal BOD, fused in‐frame at the 5′ end to the *N*‐terminal region of the *Pseudomonas syringae* INPNC. The fusion protein is transcriptionally regulated by a lac‐derived orthogonal IPTG‐inducible T5 promoter, upstream of a Shine–Dalgarno consensus ribosome binding site (RBS) optimized for *E. coli* translation kinetics. The synthetic operon includes a strong terminator downstream to ensure transcriptional insulation and reduce read‐through into plasmid backbone genes. The INPNC fusion preserves the transmembrane β‐barrel anchoring domain and the repetitive hydrophilic *C*‐terminal tandem repeats, which mediate extracellular water structuring. The vector backbone encodes an ampicillin resistance driven by a constitutive promoter for plasmid selection. The final construct enables export of the fusion protein via the Sec‐dependent pathway, guided by an *N*‐terminal signal peptide, resulting in topological orientation with the catalytic domain of BOD exposed to the extracellular environment. Post‐translationally, the apoprotein undergoes site‐specific Cu^2^⁺ chelation at the trinuclear T2/T3 and mononuclear T1 sites, generating the catalytically competent holoenzyme. The fusion construct retains a *C*‐terminal His₆‐tag for antibody‐based detection (Figures S11 and S12, Supporting Information). This genetic configuration allows for fine‐tuned, inducible expression, spatial localization, membrane integration, and functional maturation of a complex redox metalloenzyme on a bacterial platform, demonstrating a full stack of synthetic biological control from promoter to phenotype. Finally, from a system engineering perspective, this work demonstrates that catalytic phenotype—activity, selectivity, localization, and regeneration—can be genetically encoded without altering the enzyme properties. The resulting living biointerface acts as both a scaffold and an active element, enabling the integration of biological systems into abiotic energy conversion platforms. Future iterations may exploit multigene operons for cofactor biosynthesis, incorporate synthetic membrane domains for electrostatic tuning, or evolve redox enzyme libraries in situ via genotype–phenotype linkage on the cell surface.

In terms of cofactor reconstitution, our system successfully performed extracellular apo‐to‐holo conversion through post‐overexpression Cu^2^⁺ chelation, without the presence of the traditional intracellular copper chaperones (e.g., CopZ). This metalation strategy ensured robust active copper site assembly directly, which was confirmed by activity assay toward the bilirubin oxidation.

The selective water oxidation observed in BOD‐*E. coli* ELM integrates modular synthetic operons, orthogonal promoter control, and INPNC‐directed surface localization to create a membrane‐tethered redox enzyme able to catalyze water oxidation at near‐zero overpotential (27 mV). In contrast to the traditional O_2_‐reducing behavior of MCOs, the engineered BOD‐*E. coli* exclusively catalyzes the WOR, a rare inversion of native enzymatic function. In addition, the achieved faradaic current density (0.50 mA cm^−^^2^ at 1.10 V), faradaic efficiency (≈77%), and estimated TOF of 49.8 s^−1^ places this system among the top‐performing (bio)electrocatalysts for WOR under mild conditions.^[^ ^12^, ^15^, ^36^, ^37^ ^]^

The INP *C*‐terminal extracellular domain introduces a highly hydrophilic surface layer, as is well‐established for the class of proteins from which the sequence is derived. Though there are several factors thought to contribute to the ice nucleation activity of these proteins, an important factor is their ability to interact with and organize water molecules at the cell surface to support their conversion to ice.^[^ ^20^, ^21^, ^22^, ^23^ ^]^ With the established knowledge on the interaction between INPs and water molecules, we hypothesize that the incorporation of this sequence with the BOD increases local water activity. This effect, in turn, can facilitate PCET toward the formation of high‐valent copper‐oxo intermediates—essential for O─O bond formation.^[^ ^14^, ^15^ ^]^ This is in stark contrast with the catalytic behavior of purified BOD (Figure S10, Supporting Information), which under identical buffer conditions shows a significant O_2_ reduction signal and a reversible O_2_/H_2_O catalytic reaction.^[^ ^15^ ^]^

Electrochemical comparison of Tafel slopes further demonstrates the distinct catalytic behavior of BOD‐*E. coli* versus purified enzyme. The BOD‐*E. coli* system presents a Tafel slope of 250 mV dec^−1^, comparable to inorganic Cu‐based electrocatalysts operating at pH 13–14 (Table S8, Supporting Information). In contrast, the purified enzyme shows a Tafel slope of 98 mV dec^−1^, indicative of faster electron transfer but lower catalytic selectivity. This suggests that while direct electron transfer efficiency is slightly reduced in the cell‐anchored system due to increased tunneling distances or partial insulation from the membrane, this limitation is offset by superior substrate availability and local electrochemical environment. Interestingly, chronoamperometric analysis shows water oxidation by BOD‐*E. coli* proceeds with a 70 fold higher apparent diffusion coefficient for water compared to the purified BOD‐electrode system. This enhancement likely results from increased substrate (water) mass transfer driven by the hydration‐channeling effect of the INP repeat motifs,^[^ ^34^, ^35^ ^]^ previously shown to coordinate ice nucleation through ordered water alignment.^[^ ^33^, ^34^, ^35^ ^]^ This bioengineered hydration layer likely mimics the role of protons in the oxygen‐evolving complex, providing a functional analog to thylakoid lumenal dynamics.

Importantly, unlike purified BOD, which retains significant O_2_ reduction activity at pH 7.2–9.1, BOD‐*E. coli* exhibits complete suppression of ORR. This suppression is supported by the noticeable absence of cathodic currents under O_2_‐rich conditions (air‐saturated electrolytes) with BOD‐*E. coli*. Oxygen evolution was additionally confirmed electrochemically (Figure S15, Supporting Information), and the generation of reactive oxygen species (ROS) from the ORR was ruled out using a colorimetric indicator (Figure S16, Supporting Information). The functional unidirectionality, encoded at the membrane level, is rare among MCOs and has not been previously reported in any microbial cell surface‐displayed system. Such directional selectivity can be explained thermodynamically by the increased local water concentration shifting the ORR/WOR redox equilibrium.

Based on that, the genetically engineered BOD‐*E. coli* described in this study shows significant potential for integration into artificial photosynthesis platforms, particularly in the development of biohybrid systems for solar‐driven fuel production. The selective water oxidation activity enabled by surface display of BOD could be coupled with semiconductor photoanodes or biological CO_2_‐reducing catalysts to create modular, living systems capable of mimicking natural photosynthesis. However, the practical implementation of such systems at scale presents several challenges. These include maintaining long‐term operational stability (for weeks) of the engineered microbial interface, ensuring efficient and directional electron transfer under light irradiation, and achieving compatibility with scalable materials and architectures. Future efforts can be focused on these bottlenecks and exploring co‐immobilization strategies with light‐harvesting or CO_2_‐reducing components to advance this approach toward sustainable solar‐to‐chemical conversion technologies.

## 3 Conclusion

Through the integration of synthetic promoter tuning, modular operon design, and cell surface enzyme anchoring via INPNC domains, we establish a multi‐layered genetic framework that governs not only enzyme overexpression but also spatial localization, orientation, and metallocofactor maturation. The resulting ELM performs selective water oxidation with near‐zero overpotential, complete suppression of ORR, and enhanced substrate diffusion—phenotypes emerging directly from genome‐level programming. At the core of this effect is the anchoring INP extracellular domain, repurposed from its native role in ice nucleation to act as a hydration‐structuring matrix at the cell‐electrode interface. This genetically encoded hydration channel modulates PCET dynamics and shifts the catalytic equilibrium in favor of WOR. The demonstration that microbial surface architecture can drive enzyme redox selectivity challenges the prevailing understanding of MCO behavior and enables novel bioelectrochemical designs. Beyond catalysis, this work emphasizes microbial membranes not only as a barrier, but also as a scaffold for artificial photosynthesis systems.

## 4 Experimental Section

### Plasmid Design and Synthetic Operon Architecture

A modular synthetic operon was designed for the heterologous expression of a codon‐optimized BOD gene from *Myrothecium verrucaria* in *Escherichia coli*. a pSKB3 vector containing the gene insert coding for the INP with the NC‐terminal fusion (INPNC: fusion of the *N*‐terminal membrane domain INPN and the *C*‐terminal extracellular domain INPC) was used. The BOD gene was placed under the control of a T5‐lacO hybrid promoter, allowing orthogonal induction with IPTG. The BOD gene (1602 base pairs, see optimized gene sequence in the Supporting Information) was N‐terminally fused in‐frame with a membrane‐anchoring motif, the INP from *Pseudomonas syringae*, with a *N*‐terminal membrane domain INP_N_ and the *C*‐terminal extracellular domain. SnapGene 6.0.2 software was used for both plasmid and primer design.

### Transformation and Over‐Expression in *E. coli*

After gene amplifications by PCR and pSKB3‐INP‐BOD plasmid construction by Gibson assembly method (described in the Supporting Information), the plasmid was transformed into *E. coli* DH5α competent cells. The transformation was verified by electrophoresis, and Sanger sequencing was performed in the gene encoding regions for INP and BOD. Then, the pSKB3‐INP‐BOD plasmid sample was transformed into BL21 competent *E. coli* cells. The apoBOD fused to INP was over‐expressed by induction with 0.5 mmol L^−1^ IPTG in LB media at 37 °C and 250 rpm for 2.5 h (optimized conditions). The over‐expression was confirmed by SDS‐Page electrophoresis. All conditions are detailed in the Supporting Information.

### Copper Catalytic Site Reconstitution and Activity Assay

Recombinant BOD was over‐expressed as an apoprotein, without the copper active site. Functional reconstitution of the trinuclear copper cluster (T2/T3) and mononuclear Type 1 (T1) copper site was achieved post‐expression by exposing the surface‐displayed apoBOD to aqueous CuSO_4_ solutions. The formation of the Cu catalytic site in the over‐expressed recombinant BOD apoprotein was investigated under different conditions of CuSO_4_ concentration (2.00–50.00 mmol L^−1^), time (24–66 h), and temperature (4‐37 °C), and in the presence and absence of ascorbate (Tables S2–S4, Supporting Information). The optimized conditions for catalytic site reconstitution were apoBOD‐*E. coli* incubation in 10.00 mmol L^−1^ CuSO_4_ in 50 mmol L^−1^ Tris‐HCl buffer (pH 8.4), for 66 h at 37 °C and 250 rpm. After the reconstitution, the cells were washed thrice with 0.2 mol L^−1^ Tris‐HCl buffer (pH 8.4) and pelleted by centrifugation at 6000 rcf and 4 °C. The supernatant was collected, and the OD_600_ value was adjusted to ≈0.10. The BOD catalytic performance was verified by the oxidation of bilirubin (0.04 mmol L^−1^) to biliverdin in 0.20 mol L^−1^ Tris‐HCl (pH 8.4), under agitation at 37 °C, monitoring by the decrease in absorbance at 440 nm using a microplate reader.

### High‐Resolution Surface Localization by Confocal Laser‐Scanning Microscope (CLSM) and Fluorescently Assisted Cell Sorting (FACS)

Surface over‐expression of the INP‐BOD system was validated via immunolabeling with anti‐His₆ and anti‐BOD antibodies. For cell staining, DAPI fluorescent dye was used. Confocal fluorescence images were recorded on a Zeiss CLSM 710 using FITC‐antibody labeled cells. Excitation was performed with an Ar laser at a wavelength of 488 nm, and emission was monitored at 493–556 nm. Following antibody labeling, cell suspensions were diluted to an OD_600_ of ≈0.01 prior to FACS measurement. FACS data was acquired on a BD FACS Melody. Excitation was performed at a wavelength of 488 nm, and the emission was monitored using a filter at 527/32 nm and a mirror at 560 LP. 100′000 events were acquired and analyzed using the Floreada.io online analysis tool (https://floreada.io/analysis).

### Electrode Modification and Electrochemical Measurements

Electrochemical measurements were performed in a Gamry 600 Reference potentiostat (Gamry Instruments, Warminster, PA, USA), using a Pt wire and an Ag/AgCl electrode (KCl saturated filling solution) as counter and reference electrodes, respectively, and carbon cloth as working electrode. Prior to the use, carbon cloth was sonicated in 70% (v/v) ethanol and water for 10 min and dried under vacuum. Then, 50 µL of active BOD‐*E. coli* suspension in phosphate solution (OD_600_ = 2.2) were dropped on carbon cloth surface (geometric area = 1 cm^2^) and dried under vacuum. The potential values were converted to values against the standard hydrogen electrode (SHE) using the formula:

(1) $$E_{\mathrm{SHE}}=E_{\mathrm{Ag}/\mathrm{AgCl}}+0.197$$

where *E*_SHE_ is the potential value in volts versus SHE and *E*_Ag/AgCl_ is the measured potential value in volts versus the Ag/AgCl electrode. All potential values are reported against SHE. The measured currents were converted to current densities (*j*) by normalizing them to the geometric area of the working electrode. All measurements were performed at room temperature and in air‐saturated 0.1 mol L^−1^ phosphate solution (pH 9.1).

### Electron Transfer Kinetics and Substrate Diffusion Analysis

For estimating Tafel slope values, Tafel plots (*E* vs log *j*) were constructed from quasi‐steady‐state voltametric curves (1 mV s^−1^), as the Tafel slope is empirically given as:

(2) η=a+blogj

where *η* is the overpotential (in V), *a* is the exchange current density (in A cm^−2^), *b* is the Tafel slope, and *j* is the current density (in A cm^−2^). Then, Tafel slope values were estimate as the slope of the fitted curve in the region at small overpotentials, where there is no limitation by mass transfer.

The substrate apparent diffusion coefficients to the BOD‐*E. coli* and mild BOD electrode surfaces were estimated based on current‐time curves, applying the Cottrell equation:

(3) $$j\left(t\right)=n\;F\;C_{0}D^{1/2}\pi^{1/2}t^{-1/2}$$

where, *j*(t) is the current density at time 𝑡 (A cm^−2^), 𝑛 is the number of electrons transferred per molecule, F is the Faraday's constant (96485 C mol^−1^), *C*_0_ is the initial bulk substrate concentration (mol cm^−3^), 𝐷 is the diffusion coefficient (cm^2^ s^−1^), *t* is time (s), and 𝜋 is the mathematical constant (≈3.1416). This equation describes the current response in a diffusion‐controlled process when a constant potential is applied to the electrode. In a plot of *j* versus *t* ^−1/2^, the slope of the linear region is proportional to *D*. From the current density‐time curves recorded with BOD‐*E. coli* and mild BOD‐modified electrodes at 1.10 V, the plots of *j* versus *t* ^−1/2^ were constructed (Figure 3H) and their slopes were calculated to be 5.1 × 10^−3^ and 7.0 × 10^−5^ A cm^−2^ s^1/2^, respectively.

## Conflict of Interest

The authors declare no conflict of interest.

## Supporting information



Supporting Information

## Data Availability

The data that support the findings of this study are available in the supplementary material of this article.
