# Supplementary material for: Genetic Surfaceome E. coli Reprogramming Enables Selective Water Oxidation
Source: Adv Mater. 2025 Aug 15;37(47):e08100. doi: 10.1002/adma.202508100 (PMC12651133; doi:10.1002/adma.202508100)
Supplement: Supplementary file 1 — Supporting Information [file ADMA-37-e08100-s001.pdf]

ADVANCED MATERIALS

Supporting Information

for *Adv. Mater.*, DOI 10.1002/adma.202508100

Genetic Surfaceome *E. coli* Reprogramming Enables Selective Water Oxidation

Graziela C. Sedenho, Jéssica C. Pacheco, Melanie Gut, Filipe C. D. A. Lima, Sunanda Dey, Frank N. Crespilho and Ariel L. Furst**

Genetic Surfaceome *E. coli* Reprogramming Enables Selective Water Oxidation

Graziela C. Sedenho[1,2], Jéssica C. Pacheco[1,2], Melanie Gut[1], Filipe C. D. A. Lima[3], Sunanda Dey[1],

Frank N. Crespilho[1]*, and Ariel L. Furst[2]*

[1] São Carlos Institute of Chemistry, University of São Paulo (USP), São Carlos, SP 13566-590, Brazil.

[2] Department of Chemical Engineering, Massachusetts Institute of Technology, Cambridge, MA 02139, United States.

[3] Federal Institute of Education, Science, and Technology of São Paulo, Matão, SP 15991-502, Brazil.

Contents

1. Plasmid and primer designs 2

2. Gene amplification by PCR 4

3. Plasmid construction and transformation into *E. coli* 6

4. BOD over-expression on *E. coli* surface 8

5. Cu catalytic site reconstitution and BOD activity assay 9

6. Confocal laser-scanning microscope (CLSM) and fluorescently assisted cell sorting (FACS) 20

7. Estimation of holo-BOD quantity immobilized on the electrode 23

8. BOD-INP hydropathy analysis 24

9. Electrochemical monitoring of O_2 generated by BOD-decorated cells 25

10. ROS detection 25

11. Computational details 26

REFERENCES 26

1. Plasmid and primer designs

The amino acids and recombinant gene sequences of BOD from *Myrothecium verrucaria* are shown below:

BOD amino acid sequence:

VAQISPQYPMFVPLPIPPVKQPRLTVTNPVNGQEIWYYEVEIKPFTHQVYPDLGSADLVG
DGMSPGPTFQVPRGVETVVRFINNAEAPNSVHLHGSFSRAAFDGWAEDIEPGSFKDYYYPNR
QSARTLWYHDHAMHITAENAYRGQAGLYMLTDPAEDALNLPSGYGEFDIPMILTSKQYTAN
GNLVTTNGELNSFWGDVIHVNGQPWPFKNVEPRKYRFRFLDAAVSRSFGLYFADTDAIDTRL
PFKVIASDSGLLEHPADTSLLYISMAERYEVVFDFSDYAGKTIELRNLGGSIGGIGTDTDYDNT
DKVMRFVVADDTTQPDTSVVPANLRDVPFPSPTTNTPRQFRFGRTGPTWTINGVAFADVQNR
LLANVPVGTVERWELINAGNGWTHPIHIHLVDFKVISRTSGNNARTVMPYESGLKDVVWLG
RRETVVVEAHYAPFPGVYMFHCHNLIHEDHDMMAAFNATVLPDYGYNATVFVDPMEELW
QARPYELGEFQAQSGQFSVQAVTERIQTMAEYRPYAAADE

BOD recombinant gene sequence:

GTGGCGCAGATTAGCCCGCAGTATCCGATGTTTACCGTGCCGCTGCCGATTCCGCCG
GTGAAACAGCCGCGCCTGACCGTGACCAACCCGGTGAATGGCCAAGAAATTTGGTATTA
TGAAGTGGAAATTAAACCGTTTACCCATCAAGTGTATCCGGATCTGGGCAGCGCGGATCT
GGTGGGCTATGATGGCATGAGCCCGGGCCCGACCTTTCAAGTGCCTCGCGGAGTGGAAA
CCGTGGTGCGATTTATTAACAACGCGGAAGCGCCGAACAGCGTGCATCTGCATGGCAGC
TTTTCCCGCGCGGCGTTTGACGGCTGGGCGGAAGATATTACCGAACCGGGCAGCTTTAAA
GATTATTATTATCCGAACCGTCAGAGCGCGCGCACCCTGTGGTATCATGATCATGCGATG
CATATTACCGCGGAAAACGCGTATCGCGGCCAAGCGGGCCTGTATATGCTGACCGATCC
GGCGGAAGATGCGCTGAACCTGCCGAGCGGCTATGGCGAATTTGATATTCCGATGATTCT
GACGAGCAAACAGTATACCGCGAACGGCAACCTGGTGACCACCAACGGCGAACTGAACA
GCTTTTGGGGCGATGTGATTCATGTGAACGGTCAGCCGTGGCCGTTTAAAAACGTGGAAC
CGCGCAAATATCGCTTTCGCTTTCTGGATGCGGCGGTGAGCCGCAGCTTTGGCCTGTATTT
TGCGGATACCGATGCGATTGATACCCGCCTGCCGTTTAAAGTGATCGCGAGCGATAGCGG
CCTGCTGGAACATCCGGCGGATACGAGCCTGCTGTATATTAGCATGGCGGAACGCTATGA
AGTGGTGTTTGATTTTAGCGATTATGCGGGCAAAACCATTGAACTGCGCAACCTGGGCGG
CAGCATTGGCGGCATTGGCACCGATACCGATTATGATAACACCGATAAAGTGATGCGCTT
TGTGGTGGCGGATGATACCACGCAGCCGGATACGAGCGTGGTGCCGGCGAACCTGCGCG
ATGTGCCGTTTCCGAGCCCGACCACCAACACCCCGCGTCAGTTTCGCTTTGGCCGCACCG
GCCCGACCTGGACCATTAACGGCGTGGCGTTTGCGGATGTGCAGAACCGCCTGCTGGCG
AACGTGCCGGTGGGCACCGTGGAACGCTGGGAACTGATTAACGCGGGCAACGGCTGGAC
CCATCCGATTCATATTCATCTGGTGGATTTCAAGGTGATAAGCCGTACGAGCGGCAACAA
CGCGCGCACCGTGATGCCGTATGAAAGCGGCCTGAAAGATGTGGTGTGGCTGGGCCGCC
GCGAGACTGTGGTGGTGGAAGCGCATTATGCGCCGTTTCCGGGCGTGTATATGTTTCATT
GCCATAACCTGATTCATGAAGATCATGATATGATGGCGGCGTTTAACGCGACAGTCCTGC

CGGATTATGGCTATAATGCCACGGTGTTTGTGGATCCGATGGAAGAACTGTGGCAAGCGC
GCCCGTATGAACTGGGCGAATTTCAAGCGCAGAGCGGTCAGTTTAGCGTGCAAGCGGTG
ACCGAACGCATTCAGACCATGGCGGAATATCGCCCGTATGCGGCGGCGGATGAA

For the plasmid construction, a pSKB3 vector containing the gene insert coding for the ice nucleation protein with the *NC*-terminal fusion (INPNC: fusion of the *N*-terminal membrane domain INPN and the *C*-terminal extracellular domain INPC) was used, as previously reported for the expression of estrogen receptor alpha protein.[1] INP is an anchor protein that displays non-specific products, such as other proteins, on the surface of *E. coli*. The pSKB3 vector containing the gene inserts coding for the INP and BOD is referred to as pSKB3-INP-BOD.

Though alternative surface expression peptides have been employed for the surface display of proteins on *E. coli*, many of these sequences are challenging to deploy due to their cytotoxicity in the host organism, which leads to inconsistent and low-yielding induction and expression on the cell surface. To date, our lab has evaluated two additional surface expression tags (OmpA and LPP). Consistent challenges with overexpression using our standard pSKB3 vector were seen across five proteins evaluated (*unpublished results*). These observations are consistent with literature reports that surface-expressed proteins for display on *E. coli* can cause toxicity and are limited by the size of the proteins to be expressed.[2–5] The advantages of surface expression have been further established in the cost analysis of the production of surface-expressed proteins. Based on cost analyses, our surface-expressed system yields scaffolded proteins that cost at least an order of magnitude less than purified proteins.

Before the insertion of the insert (BOD gene, 1,602 bp) into the backbone (pSKB3-INP gene, 6,745 bp), the genes were amplified by polymerase chain reaction (PCR). For this, the primers before and after the insert and backbone regions in the plasmid gene sequence were designed, as shown in Figure S1. All primers contain 35-38 nucleotides of homology between the insert and backbone regions. The primers are divided into two regions: “priming” and “overlap” regions. The priming regions of the backbone primers showed 65 and 69% GC content and the annealing temperature of 69 °C, whereas the insert primers showed 67 and 69% GC and the annealing temperature equals to 71 °C. The annealing temperatures were calculated using Tm Calculator 1.15.0. (New England BioLabs Inc.), at 500 nM primer concentration and Q5 High-Fidelity DNA Polymerase.

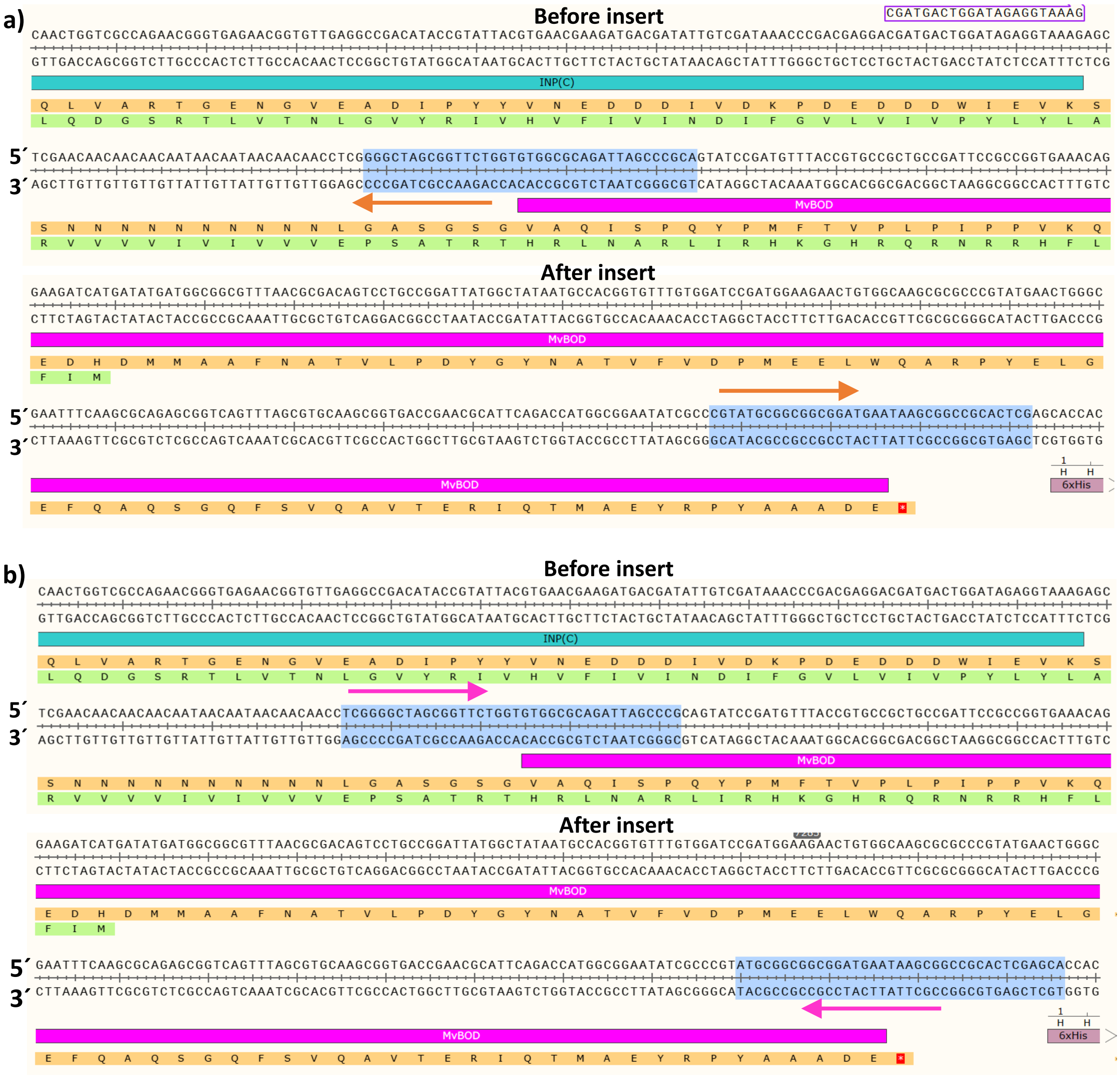

Figure S1. Designed primers for the backbone (a) and insert (b) highlighted in blue in the plasmid gene sequence. Gene sequence reproduced from SnapGene 6.0.2.

2. Gene amplification by PCR

The backbone and insert genes were amplified by PCR using the designed primers. The reactions were performed in a total volume of 25 µL containing 1X Q5 High-Fidelity DNA Polymerase, 0.5 µmolL^{-1} of each primer, and 5 ng of the insert or backbone gene. The PCR steps are summarized in Table S1. The gel electrophoresis of the insert PCR product showed a band at approximately 1,500 bp, which corresponds to the BOD gene, and the backbone PCR product presented a band between 8,000 and 6,000 bp, as expected for pSKB3-INP (Figure S2). The band at a very high base pair count indicates that some self-annealing occurred during the backbone amplification.

Table S1. PCR conditions for amplification of BOD (insert) and pSKB3-INP (backbone) genes.

Step	Temperature / ºC	Time
1. Initial denaturation	98.0	3 min
2. Denaturation	98.0	30 s
3. Annealing	69.0 (backbone), 7.01 (insert)	30 s
4. Extension	72.0	4 min
Repeat steps #2-4 for 35 times		
5. Final extension	72.0	10 min

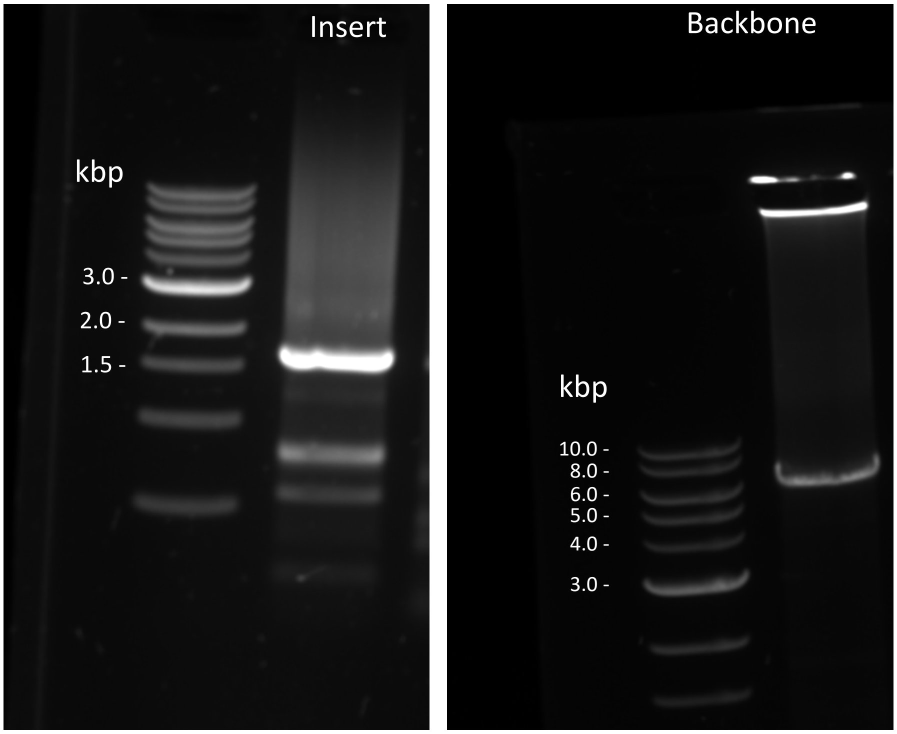

Figure S2. Gel electrophoresis of insert and backbone PCR products. Conditions: 10 $mgmL^{-1}$ agarose gel, 100 V for 30 min.

The amplified gene samples were purified before use. The resulting PCR backbone solution was first digested with DnpI enzyme. This enzyme specifically cleaves GATC sites if the adenine is methylated. Methylation does not occur during PCR amplification, but only in cells. Therefore, this procedure was used to digest cell-derived DNA left after PCR. Next, the amplified backbone solution after digestion and the PCR insert solution were purified using a silica spin-column to remove the enzymes used in molecular biology and double- or single-stranded PCR products smaller than 100 bases or higher than

10,000 bases. After purification, the concentrations of the amplified BOD and pSKB3-INP gene solutions were determined to be 37.3 and 26.4 ngμL^{-1}, respectively (Absorbance (A) at 260 nm; Figure S3). The ratios between the absorbance values at 260 and 280 nm (A_{260}/A_{280}), and 260 and 230 nm (A_{260}/A_{230}) were higher than 1.8 indicating that the DNA samples were of high purity.

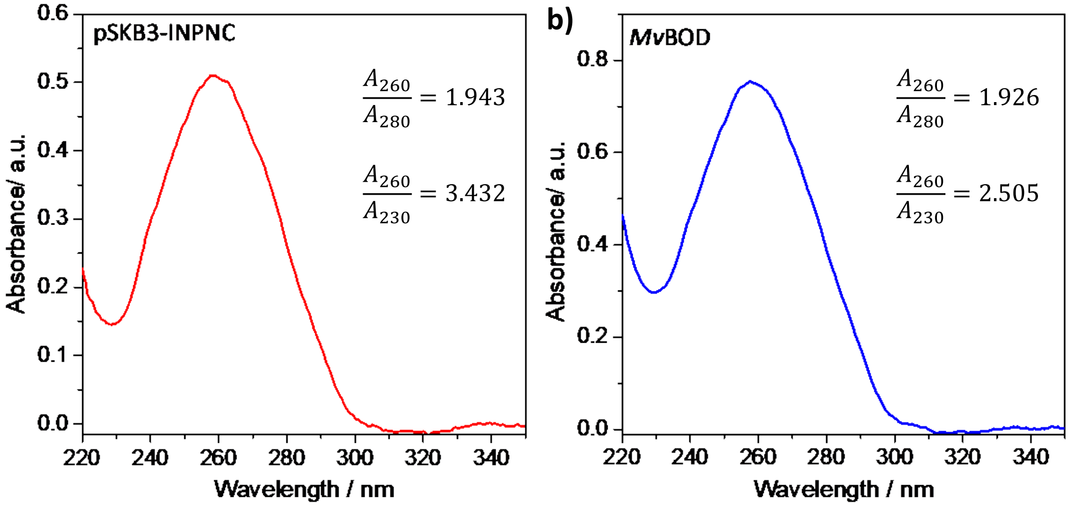

Figure S3. UV-vis spectra of the amplified and purified pSKB3-INP (a) and BOD (b) gene solutions.

3. Plasmid construction and transformation into *E. coli*

The pSKB3-INP-BOD plasmid was then constructed by Gibson assembly. This is a molecular cloning method that allows for the joining of multiple DNA fragments by an isothermal reaction. BOD and pSKB3-INP DNA solutions (at a 1:3 ratio) were mixed with three enzymes to promote the ligation: (i) 5′ exonuclease, which degrades the 5′ end sequences and exposes the complementary sequence for annealing; (ii) 3′ extension activity of a DNA polymerase to fill in the gaps on the annealed regions; and (iii) DNA ligase, to seal the nick and covalently link the DNA fragments together. The reaction was performed at 50 °C for 1 h.

Next, the pSKB3-INP-BOD plasmid was transformed into *E. coli* DH5α competent cells. Competent cells were added to the ligation mixture and grown in culture media at 37 °C for 1 h at 250 rpm. Then, 100 μL of the suspension was spread on agar plates containing 50 mgL^{-1} kanamycin and cultivated overnight at 37 °C. A control experiment was performed using the backbone DNA solution rather than the ligation mixture. Figure S4a shows 18 and 22 *E. coli* DH5α colonies on the plates after the transformation, whereas the control plates displayed only 1 colony each (Figure S4b).

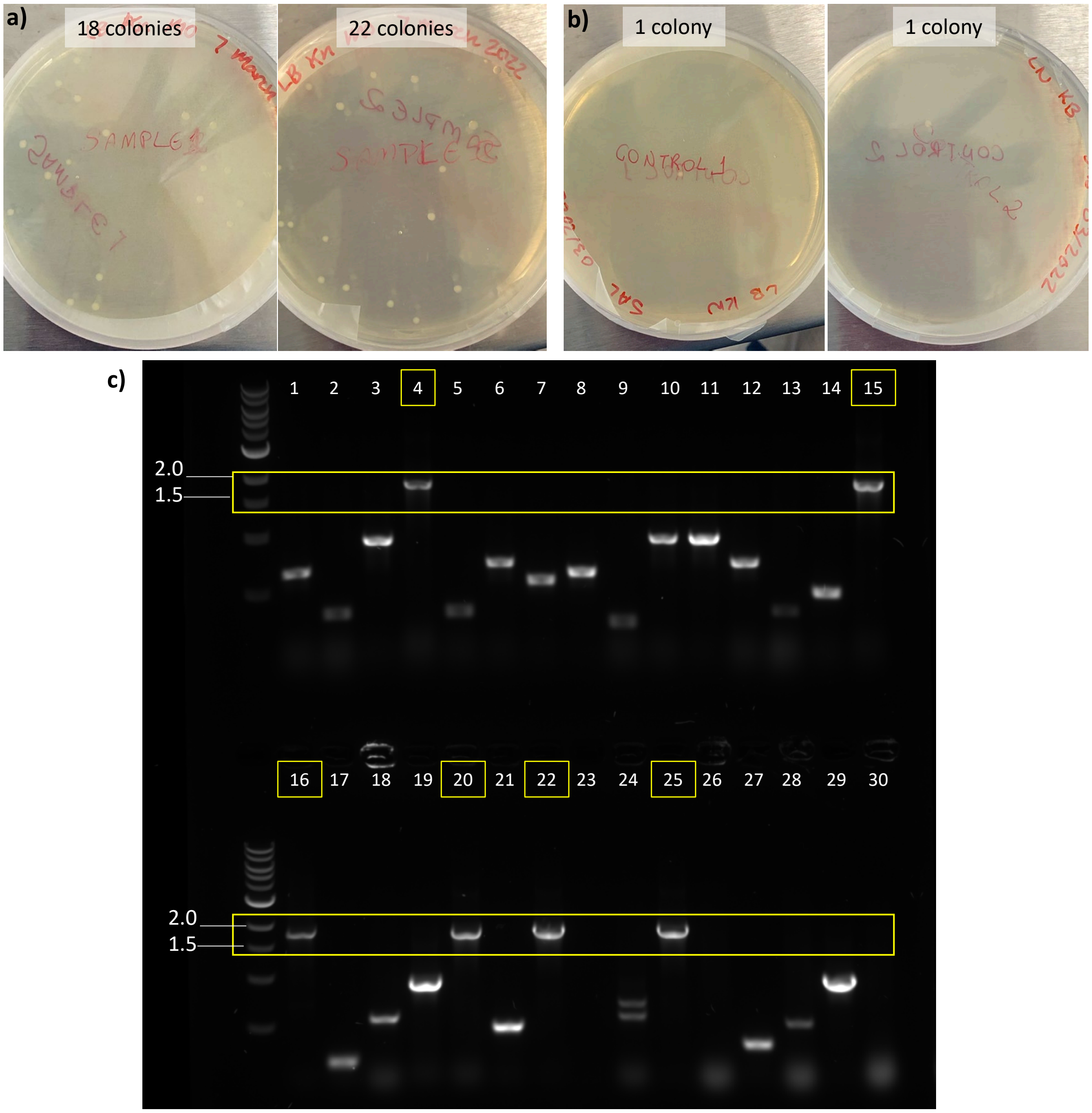

Figure S4. Agar plates (a) after the pSKB3-INP-BOD plasmid and (b) backbone (control) transformations into *E. coli* DH5α competent cells. (c) Gel electrophoresis of *E. coli* DH5α colonies after the transformation. Conditions: 10 mgmL^{-1} agarose gel, 100 V for 30 min.

To verify the plasmid transformation into *E. coli*, samples of the colonies were collected and amplified by PCR and then analyzed by gel electrophoresis (Figure S4c). As the total base pairs of BOD and primers employed in the PCR are 1,760, the pSKB3-INP-BOD transformation into *E. coli* can be verified in colonies #4, 15, 16, 20, 22, and 25 by the presence of bands in the region between 2,000-1,500 bp. The 6 colonies with the pSKB3-INP-BOD plasmid were cultivated in sterile Luria-Bertani (LB) media containing 50 mgL^{-1} kanamycin at 37 °C and 250 rpm for 7 h, for preparing a cell stock and plasmid sequencing. Then, the plasmid was separated from the bacterial colony to obtain pure plasmid solutions. Sanger sequencing was performed on the gene encoding regions for INP and BOD. The gene sequence of the 6 samples were aligned to the designed pSKB3-INP-BOD gene for comparison (Figure S5). Samples from colonies #15, 20, and 25 showed the expected gene sequence. The plasmid

concentration in those samples was determined by UV-vis, as previously mentioned, and was 95.6, 67.0, and 82.5 ngμL^{-1}, respectively.

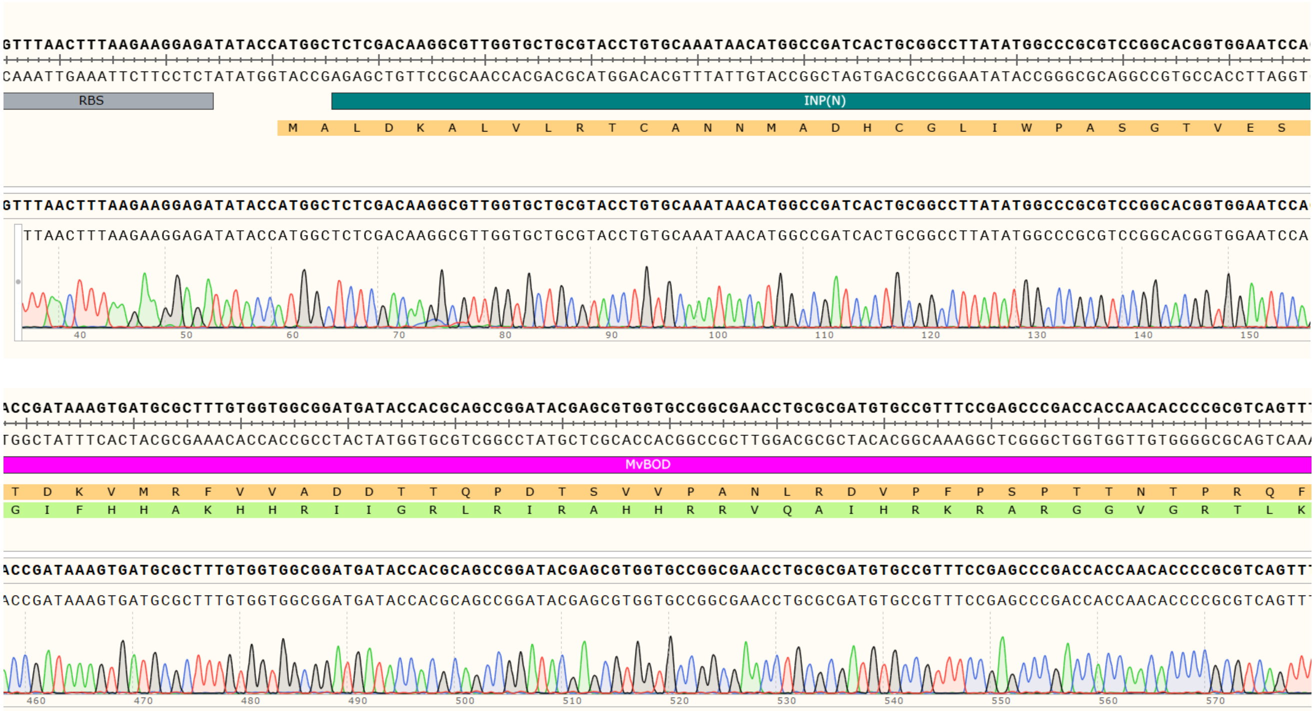

Figure S5. Alignment of INP and BOD gene encoding regions of to the designed pSKB3-INP-BOD plasmid. Software used: SnapGene 6.0.2.

4. BOD over-expression on *E. coli* surface

The pSKB3-INP-BOD plasmid sample was transformed into BL21 competent *E. coli* cells. The transformation procedure was similar to that described for *E. coli* DH5α cells. Starter cultures (5 mL of LB media containing 50 mgL^{-1} kanamycin) were grown from single colonies overnight at 37 °C and 250 rpm, and used to inoculate 20 mL of LB media. Cultures grew at 37 °C to an OD_{600} of 0.4-0.5 (approximately 1.5 h) and then, the BOD expression was induced by adding 0.5 mmolL^{-1} IPTG. Different temperatures (25, 30, and 37 °C) and times (2.5, 4.5, and 19.5 h) of incubation were studied. After the induction, an aliquot of each sample was lysed by sonication and analyzed by SDS-PAGE electrophoresis to verify the protein expression (Figure S6). Electrophoresis was performed using a 4-12% Tris-glycine gel, at 125 V for 1h 15 min.

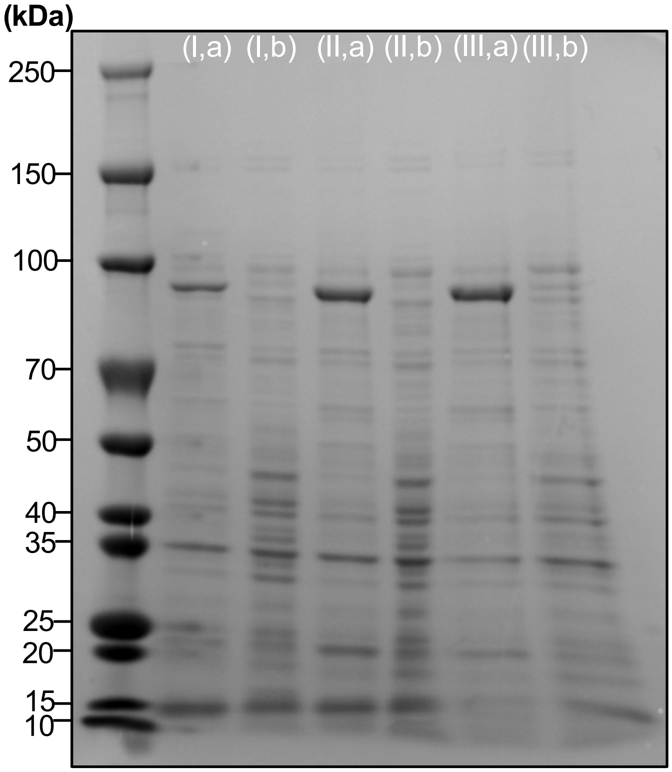

Figure S6. C) Blue Coomassie-stained SDS-Page gel of lysed *E. coli* after INP-BOD over-expression at 25 (I), 30 (II), and 37 °C (III) in the absence (control, a) and presence of inducer (IPTG, b).

5. Cu catalytic site reconstitution and BOD activity assay

The formation of the Cu catalytic site in the over-expressed recombinant BOD apoprotein was investigated under different conditions, based on previous reports.[6–8] The success of the catalytic site formation was analyzed by the enzyme's homogeneous biocatalytic activity toward the oxidation of bilirubin into biliverdin. Bilirubin, the natural enzyme substrate, is oxidized to biliverdin, and its consumption can be measured by the absorbance decrease at 440 nm.

Two main strategies were investigated for the formation of the Cu catalytic site: (i) over-expression in the presence of Cu^{2+} and (ii) Cu^{2+} chelation after over-expression. For each method, different Cu^{2+} and IPTG concentrations, temperatures, and periods were investigated. The BOD over-expression in each condition was analyzed by SDS-PAGE electrophoresis. The Cu^{2+} chelation after the induction was performed in 50 mmol L^{-1} Tris buffer (pH 7.6).

The activity assay was performed in presence of 0.04 mmolL^{-1} bilirubin in 0.20 mol L^{-1} Tris-HCl (pH 8.4), under agitation at 37 °C, by using a microplate reader. The conditions of expression and Cu^{2+} chelation, as well as the results of the activity assay, are shown in Tables S2 and S3.

Table S2. Induction conditions in presence of Cu^{2+} and the respective BOD over-expression and activity assay results.

**Induction conditions**					
IPTG concentration / $mmolL^{-1}$	$CuSO_4$ concentration / $mmolL^{-1}$	Temperature / ^{o}C	Time / h	**Over expressed?**	**Active?**
0.5	0.25	25	19	No	N.a.
0.5	0.25	30	5	Yes	No
0.5	0.25	37	3	Yes	No
0.25	2.00	37	3	Yes	No
0.25	5.00	37	3	Yes	No
0.25	2.00	18	16	Yes	No
0.15	2.00	30	15	Yes	No

Table S3. Cu^{2+} chelation conditions after the BOD over-expression and the activity assay result.

**Cu^{2+} chelation conditions**				
Temperature / ^{o}C	Time / h	$CuSO_4$ concentration / $mmolL^{-1}$	Ascorbate concentration / $mmolL^{-1}$	**Active?**
4	66	2.00	50.00	No
4	66	2.00	0	No
4	66	10.00	50.00	No
4	66	10.00	0	No
4	66	50.00	0	No
25	66	2.00	50.00	No
25	66	2.00	0	No
25	66	10.00	50.00	No
25	66	10.00	0	Yes

The sample over-expressed in absence of Cu^{2+} and subsequently submitted to chelation in presence of 10.00 $mmolL^{-1}$ $CuSO_4$, for 66 h at 25 ^{o}C showed catalytic activity towards bilirubin oxidation reaction. Bilirubin shows a maximum absorbance at 440 nm (Figure S7a), with extinction coefficient of 56.3 $mM^{-1}cm^{-1}$. The decrease in absorbance at 440 nm with reaction time evidences the bilirubin oxidation catalyzed by the over-expressed BOD on the *E. coli* surface (Figure S7b), indicating the proper formation of the BOD Cu site. For comparison, a similar assay was performed with commercially

available BOD (from Sigma Aldrich), as shown in Figure S7c. Control experiments were performed in the absence of enzyme. The slope of bilirubin consumption curve for over-expressed and commercially available BOD are (-4.87 ± 0.13) × 10^{-3} and (-39.04 ± 6.37) × 10^{-3} min^{-1}, respectively.

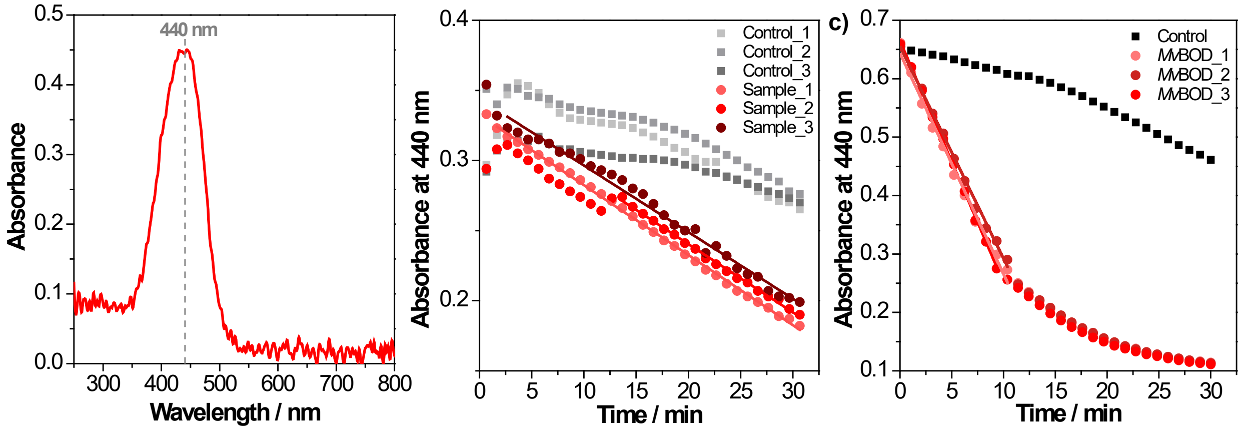

Figure S7. a) Absorption spectrum of 0.04 mmolL^{-1} bilirubin in 0.20 molL^{-1} Tris-HCl (pH 8.4) solution. Bilirubin consumption kinetics through the absorbance at 440 nm measurement with time, using (b) over-expressed enzyme in presence of 10 mmolL^{-1} $CuSO_4$, at 25 °C for 66 h and (c) commercially available BOD.

Based on this result, the Cu^{2+} chelation conditions were optimized, as presented in Table S4. Results of the BOD activity assay indicate that the Cu catalytic site is not formed or formed with very low yield over 24 and 44 h of incubation in presence of $CuSO_4$, whereas incubation for 66 h produces active BOD. The highest bilirubin oxidation homogeneous kinetics was obtained after apoBOD-*E. coli* incubation in 10.00 mmol L^{-1} $CuSO_4$, for 66 h at 37 °C, as shown in Figure 1C. The slope of bilirubin consumption curve for this sample was (-18.85 ± 1.31) × 10^{-3} min^{-1}. The effect of the incubation Cu^{2+} concentration and temperature for 66 h on the kinetics of the bilirubin oxidation can be visualized in Figure S8.

Table S4. Studied BOD Cu^{2+} chelation conditions.

**Cu^{2+} chelation conditions**			**Active?**
Temperature / °C	Time / h	$CuSO_4$ concentration / mmolL^{-1}	
4	24	5.00	No
		10.00	No
		20.00	No
	44	5.00	No
		10.00	No
		20.00	No

		5.00	Yes
	66	10.00	Yes
		20.00	Yes
		5.00	No
	24	10.00	No
		20.00	No
		5.00	No
25	44	10.00	No
		20.00	No
		5.00	Yes
	66	10.00	Yes
		20.00	Yes
		5.00	No
	24	10.00	No
		20.00	No
		5.00	No
30	44	10.00	No
		20.00	No
		5.00	Yes
	66	10.00	Yes
		20.00	Yes
		5.00	No
	24	10.00	Yes (low)
		20.00	Yes (low)
		5.00	No
37	44	10.00	No
		20.00	Yes (low)
		5.00	Yes
	66	10.00	Yes
		20.00	Yes

Inductively Coupled Plasma Mass Spectrometry (ICP-MS) measurements to determine the [Cu^{2+}] bound to the cell surface were conducted at the core facilities of the Center for Environmental Health Sciences (CEHS) at MIT. An Agilent 7900 ICP-MS system in gas-mode was used for all measurements. Induced and uninduced cell samples (OD_{600} = 0.2, 1 mL) were exposed to 10.00 mmol L^{-1} $CuSO_4$, for 24, 48, and 66 h at 25 °C and subsequently washed (3 × PBS). Cell pellets (OD_{600} = 20,10 µL) were lysed in HNO_3 (70%, 990 µL) overnight and diluted 1:30 into 2% HNO_3 to a final volume of 2 mL. Germanium was added as an internal standard, and all samples were filtered using 0.22 µm cellulose acetate filters (Thermo Scientific) prior to ICP-MS analysis. Samples were run in biological triplicates. The results are shown in Tables S5-S7.

Table S5. Fe and Cu concentrations of lysates from BOD overexpressing cells determined by ICP-MS.

				72 Ge (ISTD) [He-mode]		56 Fe [He-mode]				63 Cu [He-mode]			
**Incubation time / h**	**Induced**	**Cu^{+2} treaded**	**Replicate**	**CPS**	**CPS RSD**	**Conc. / ppb**	**Conc. RSD / ppb**	**CPS**	**CPS RSD**	**Conc. / ppb**	**Conc. RSD / ppb**	**CPS**	**CPS RSD**
0			A		1.08	<0.00000	N/A	49484.32	1.76	<0.00000	N/A	6787.36	2.91
	+	+	B	282760.20	1.39	<0.00000	N/A	52842.83	2.21	<0.00000	N/A	9295.47	0.85
			C	280199.80	1.61	<0.00000	N/A	50732.88	2.38	<0.00000	N/A	6739.57	1.47
			A	272639.71	0.39	<0.00000	N/A	49126.46	0.78	<0.00000	N/A	6995.25	2.08
	-	-	B	287461.10	0.65	<0.00000	N/A	47641.47	1.27	<0.00000	N/A	4572.00	0.54
			C	263363.31	0.85	<0.00000	N/A	48587.10	1.52	<0.00000	N/A	4545.34	0.88
24			A	271084.28	0.87	<0.00000	N/A	49265.73	1.14	0.69	2.33	18867.17	1.33
	+	+	B	269568.02	1.11	<0.00000	N/A	44988.79	1.40	0.66	0.77	18448.84	1.37
			C	275108.58	0.30	10.21	0.57	163436.84	0.06	0.94	0.85	21570.98	0.59
			A	194409.80	1.33	<0.00000	N/A	48785.20	1.45	<0.00000	N/A	4622.02	2.18
	-	-	B	267637.72	0.53	<0.00000	N/A	65409.82	1.10	<0.00000	N/A	5037.70	0.78
			C	261899.01	1.26	<0.00000	N/A	49396.18	2.04	<0.00000	N/A	4739.83	4.67
48			A	268702.35	0.36	<0.00000	N/A	44401.47	1.14	2.78	3.61	38822.52	2.14
	+	+	B	270225.92	0.97	<0.00000	N/A	42640.58	2.07	2.37	0.24	35057.46	1.11
			C	260779.83	0.89	<0.00000	N/A	51057.65	1.45	2.56	1.65	35604.32	1.21
			A	260686.02	0.45	<0.00000	N/A	48314.91	1.57	<0.00000	N/A	4645.35	2.05
	-	-	B	273251.50	0.35	<0.00000	N/A	48180.98	1.14	<0.00000	N/A	4325.25	2.77
			C	272814.72	0.94	<0.00000	N/A	48826.16	0.76	<0.00000	N/A	4534.19	2.72
66			A	265079.65	1.37	<0.00000	N/A	42737.57	2.20	5.85	1.26	67532.31	0.37
	+	+	B	281402.20	0.14	<0.00000	N/A	39786.94	0.28	5.49	1.32	68077.99	1.03
			C	270748.53	1.26	<0.00000	N/A	39988.78	0.29	5.80	0.03	68503.70	1.27
			A	263510.21	0.65	<0.00000	N/A	49056.34	1.17	<0.00000	N/A	4628.68	3.59
	-	-	B	249072.87	0.53	<0.00000	N/A	55477.93	0.78	<0.00000	N/A	4690.92	3.77
			C	268185.05	0.70	<0.00000	N/A	50341.44	0.57	<0.00000	N/A	4748.72	3.80

Table S6. Mg and Al concentrations of lysates from BOD overexpressing cells determined by ICP-MS.

				72 Ge (ISTD) [He-mode]		24 Mg [He-mode]				27 Al [He-mode]			
Incubation time / h	Induced	Cu^{+2} treaded	Replicate	CPS	CPS RSD	Conc. / ppb	Conc. RSD / ppb	CPS	CPS RSD	Conc. / ppb	Conc. RSD / ppb	CPS	CPS RSD
0	+	+	A	294477.61	1.08	<0.00000	N/A	12012.96	1.82	15.59	4.10	16414.00	2.12
			B	282760.20	1.39	<0.00000	N/A	12626.85	3.47	18.99	3.40	17102.54	2.81
			C	280199.80	1.61	<0.00000	N/A	12202.01	1.19	20.27	2.39	17440.73	0.83
	-	-	A	272639.71	0.39	<0.00000	N/A	13465.36	1.54	27.69	2.20	19789.20	1.13
			B	287461.10	0.65	<0.00000	N/A	13705.63	2.76	25.94	5.04	20163.03	2.18
			C	263363.31	0.85	<0.00000	N/A	13437.56	2.47	30.90	2.78	20293.26	1.72
24	+	+	A	271084.28	0.87	<0.00000	N/A	12333.24	0.68	31.50	2.94	21114.42	2.47
			B	269568.02	1.11	<0.00000	N/A	12232.04	0.96	31.29	6.51	20913.00	2.55
			C	275108.58	0.30	<0.00000	N/A	13831.27	2.29	30.66	3.05	21106.60	1.80
	-	-	A	194409.80	1.33	<0.00000	N/A	13053.80	1.50	53.80	3.94	21181.24	3.92
			B	267637.72	0.53	<0.00000	N/A	12989.38	1.98	31.79	0.55	20954.19	0.29
			C	261899.01	1.26	<0.00000	N/A	13266.28	2.48	33.93	5.22	21289.11	4.20
48	+	+	A	268702.35	0.36	<0.00000	N/A	11186.76	1.18	21.42	2.63	17158.11	1.37
			B	270225.92	0.97	<0.00000	N/A	10937.69	0.94	20.54	6.41	16920.11	2.00
			C	260779.83	0.89	<0.00000	N/A	12204.21	2.18	24.94	2.88	17930.14	1.02
	-	-	A	260686.02	0.45	<0.00000	N/A	13101.70	0.57	32.64	1.88	20716.01	1.03

66			B	273251.50	0.35	<0.00000	N/A	13106.16	1.36	30.57	2.08	20930.77	1.36
			C	272814.72	0.94	<0.00000	N/A	13703.38	0.56	30.60	4.82	20902.99	1.78
			A	265079.65	1.37	<0.00000	N/A	8001.28	0.13	1.20	13.13	9464.40	1.95
	+	+	B	281402.20	0.14	<0.00000	N/A	8128.03	1.69	<0.00000	N/A	9184.24	0.63
			C	270748.53	1.26	<0.00000	N/A	8938.53	0.70	0.72	46.94	9485.55	1.95
		-	A	263510.21	0.65	<0.00000	N/A	13431.98	0.61	29.65	1.99	19847.07	1.73
	-		B	249072.87	0.53	<0.00000	N/A	13354.20	0.39	32.86	2.95	19869.36	1.57
			C	268185.05	0.70	<0.00000	N/A	13465.40	2.53	29.41	0.46	20108.56	0.50

Table S7. Cu^{2+} concentrations of lysates from BOD overexpressing cells determined by ICP-MS and calculated Cu^{+2} per protein.

**Time / h**	**[Cu^{+2}] measured / ppb**	**Cu^{+2} total in sample / mg**	**[Cu^{+2}] / mg cell^{-1}**	**Cu^{+2} / protein**
0	<0.00	<0.00	<0.00	<0.00
24	0.76 ± 0.12	$2.29 \pm 0.37 \times 10^{7}$	$1.43 \pm 0.23 \times 10^{15}$	0.27 ± 0.04
48	2.57 ± 0.17	$7.70 \pm 0.50 \times 10^{7}$	$4.81 \pm 0.32 \times 10^{15}$	0.91 ± 0.06
66	5.71 ± 0.16	$1.71 \pm 0.48 \times 10^{7}$	$1.07 \pm 0.30 \times 10^{15}$	2.03 ± 0.06

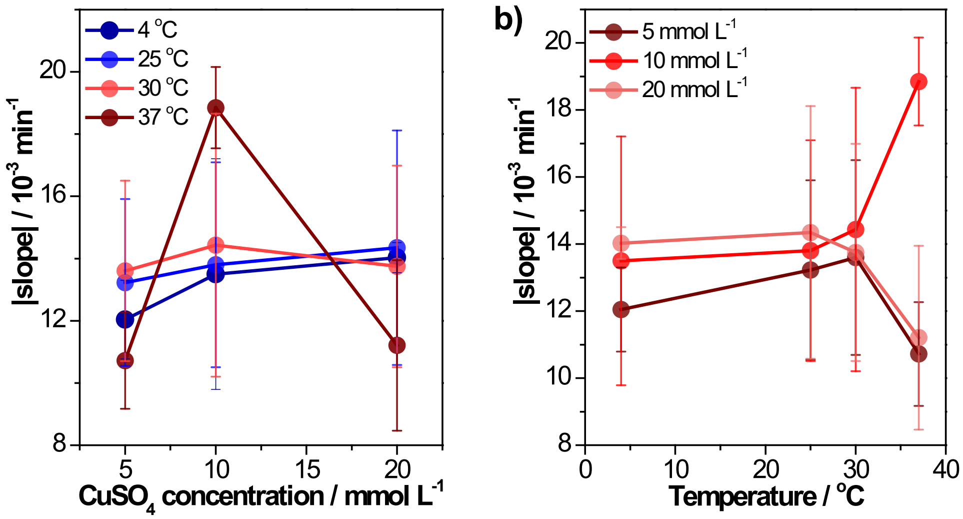

Figure S8. (a) Bilirubin consumption kinetics through the absorbance at 440 nm measurement with time, using BOD-*E. coli* after the Cu^{2+} chelation in 10.00 mmolL^{-1} $CuSO_4$, for 66 h at 37 °C (red curves) and non-induced *E. coli* cells (control, grey curves). Slopes of bilirubin consumption curves as a function of (a) $CuSO_4$ concentration and (b) temperature during the Cu^{2+} chelation.

The activity of BOD-displayed *E. coli* surface was estimated by the following equation:

$$Units/cell\,volume = \frac{\left({A_{440}}/{\mathrm{min}}^{\mathrm{sample}} - {A_{440}}/{\mathrm{min}}^{\mathrm{control}}\right) \times V_{\mathrm{total}}}{\varepsilon \times V_{\mathrm{cell}}}$$

where A_{440} is the absorbance at 440 nm, A_{440}/min ratio is the slope of the activity assay plots, V_{total} and V_{cell} are the total assay and cell suspension ($OD_{600} = 0.1$) volumes, respectively, and ε is the bilirubin extinction coefficient at 440 nm (56.3 mM^{-1}cm^{-1}). Under optimized conditions for active site formation, BOD-displayed *E. coli* surface activity was estimated to be 0.28 ± 0.02 mUmL^{-1}. The activity of commercially available BOD was estimated to be 0.64 ± 0.01 mUmL^{-1}, by the same assay.

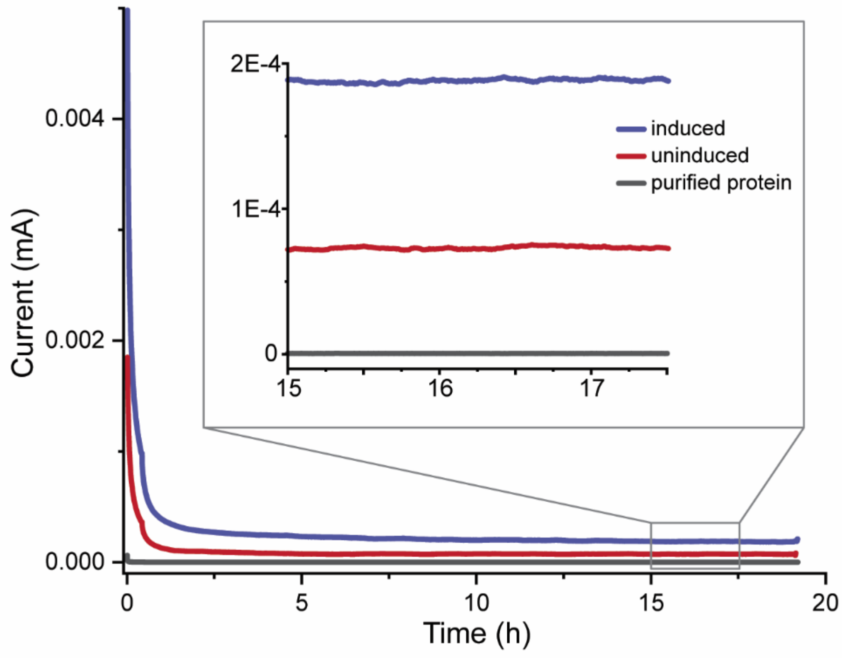

Figure S9. Current-time curves recorded at 1.10 V with BOD-*E. coli*, uninduced *E. coli* cells and mild and purified BOD immobilized on carbon cloth electrode in air-saturated 0.1 M phosphate solution (pH 9.1).

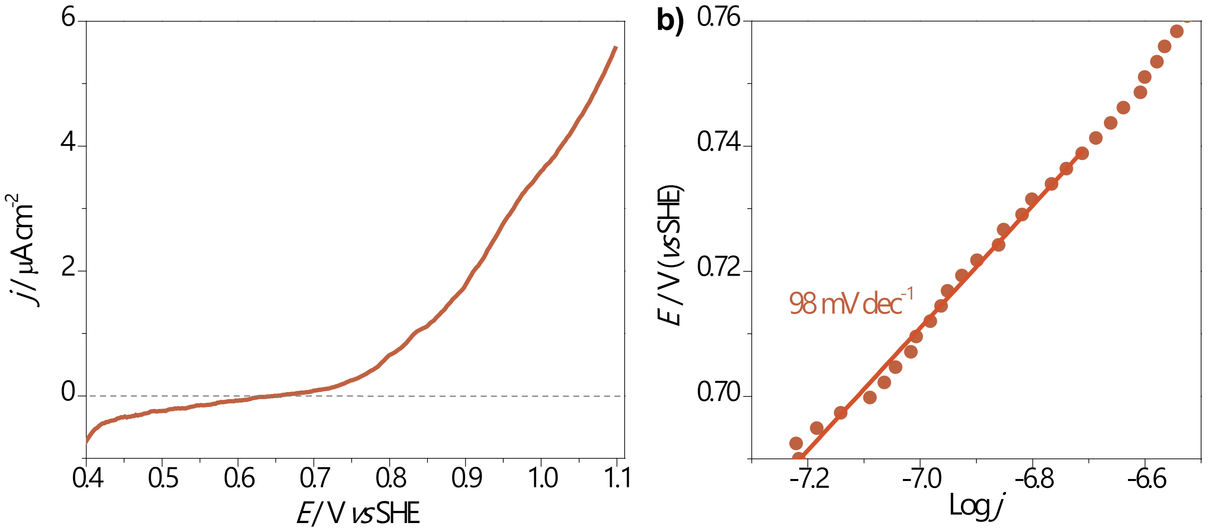

Figure S10. a) Linear voltammogram at 10 mVs^{-1} recorded with mild and purified BOD adsorbed on carbon cloth electrode in air-saturated 0.1 M phosphate solution (pH 9.1). b) Tafel plot.

Table S8. Electrochemical performance of different non-noble metal-based electrocatalysts for water oxidation reaction.

Electrocatalyst	Electrolyte	Tafel slope / $mVdec^{-1}$	TOF / s^{-1}	Overpotential / V	FE / %	Ref.
Benzimidazole Schiff base copper(II) complexes	0.1 M KOH	344 – 485	NR	0.36 – 1.07	NR	[9]
Copper–iron metal-organic frameworks	1.0 M KOH	64.8 – 92.4	NR	0.370 – 0.450	NR	[10]
Cu(II)-based bromo-salophen complexes	0.1 M KOH	187 – 432	NR	0.300	NR	[11]
Cu-based compounds on copper foam	1.0 M KOH	73 – 118	NR	0.084 – 0.240	99	[12]
CuO-based nanocomposites	1.0 M KOH	165 – 246	NR	0.200	NR	[13]
Cu-polyhistidine	0.1 M phosphate solution (pH 9.0)	NR	0.26	0.15	NR	[14]
Soluble trinuclear copper(II) complex	0.1 M phosphate solution (pH 7)	NR	3.6	0.550	45*	[15]
$Cu(TCA)_2$	0.1 M acetate solution (pH 12.5)	NR	0.6	0.29	NR	[16]
Mononuclear copper complexes of bis-amide ligands	0.1 M phosphate buffer (pH 13)	NR	10 – 1462	0.697 – 0.767	65 – 79	[17]
Fe and F co-doped CoO	1 M KOH	55.8 – 60.1	NR	0.169	NR	[18]
Defective NiFe layered double hydroxide	1.0 M KOH	58.2	0.0475	0.228	~100	[19]
Co-O-Fe bimetallic sites in nanoflower-like electrocatalyst	1.0 M KOH	54.4	0.0194	0.217	~100	[20]
Bacterial small laccase-based artificial metalloenzymes	50 mM tris-HCl (pH 8.0)	177	0.2	NR	16	[21]
BOD-*E. coli*	0.1 M phosphate solution (pH 9.1)	250	49.8	0.027	77	This work

FE: faradaic efficiency; NR: not reported; TCA: 1-Mesityl-1H-1,2,3-triazole-4-carboxylic acid; TOF: turnover frequency.
*At pH 8.

6. Confocal laser-scanning microscope (CLSM) and fluorescently assisted cell sorting (FACS)

Cells were treated either with an anti-mvBOD-1°-antibody (10 μgmL^{-1}) for 24 h, followed by an anti-rabit-2°-antibody (10 μgmL^{-1}) for 1h, or with a fluorescently labelled anti-His antibody (10 μgmL^{-1}) for 24 h prior to imaging. Additionally, cells were treated with DAPI (1 μgmL^{-1}) for 10 min prior to imaging.

Confocal fluorescence images were recorded on a Zeiss CLSM 710 using FITC-antibody and DAPI labeled cells. Excitation was performed with an Ar laser at a wavelength of 488 nm (emission monitored at 493–556 nm) for FITC, and with a 358 nm laser for DAPI (emission monitored at 450–465 nm).

Following antibody labeling, cell suspensions were diluted to an OD_{600} of ~0.01 prior to FACS measurement. FACS data was acquired on a BD FACS Melody. Excitation was performed at a wavelength of 488 nm, and the emission was monitored using a filter at 527/32 nm and a mirror at 560 LP. 100’000 events were acquired and analyzed using the Floreada.io online analysis tool (https://floreada.io/analysis).

Confocal microscopy of apoBOD-*E. coli* and BOD-*E. coli* labeled with fluorescent anti-BOD antibody were digitally processed to improve signal clarity and highlight cellular morphology. Image processing was conducted using Python 3.10 with the libraries OpenCV, Pillow (PIL), and NumPy. The original RGB image was first smoothed using a Gaussian blur filter (kernel size 5×5) to reduce high-frequency background noise while preserving cellular structure. The luminance channel was converted to grayscale and enhanced via histogram equalization to improve dynamic range and contrast. To accentuate cellular borders, Canny edge detection was applied, followed by morphological dilation (3×3 kernel) to generate a binary edge mask. This mask was merged with the smoothed image using linear blending ($\alpha = 1.0$, $\beta = 0.5$) to visually enhance fluorescence outlines. Final image brightness and contrast were adjusted using the ImageEnhance module in PIL (brightness factor: 1.2; contrast factor: 1.3) to aid in visualization. All processing steps were non-destructive and did not alter the inherent morphology or fluorescence distribution of the original cells.

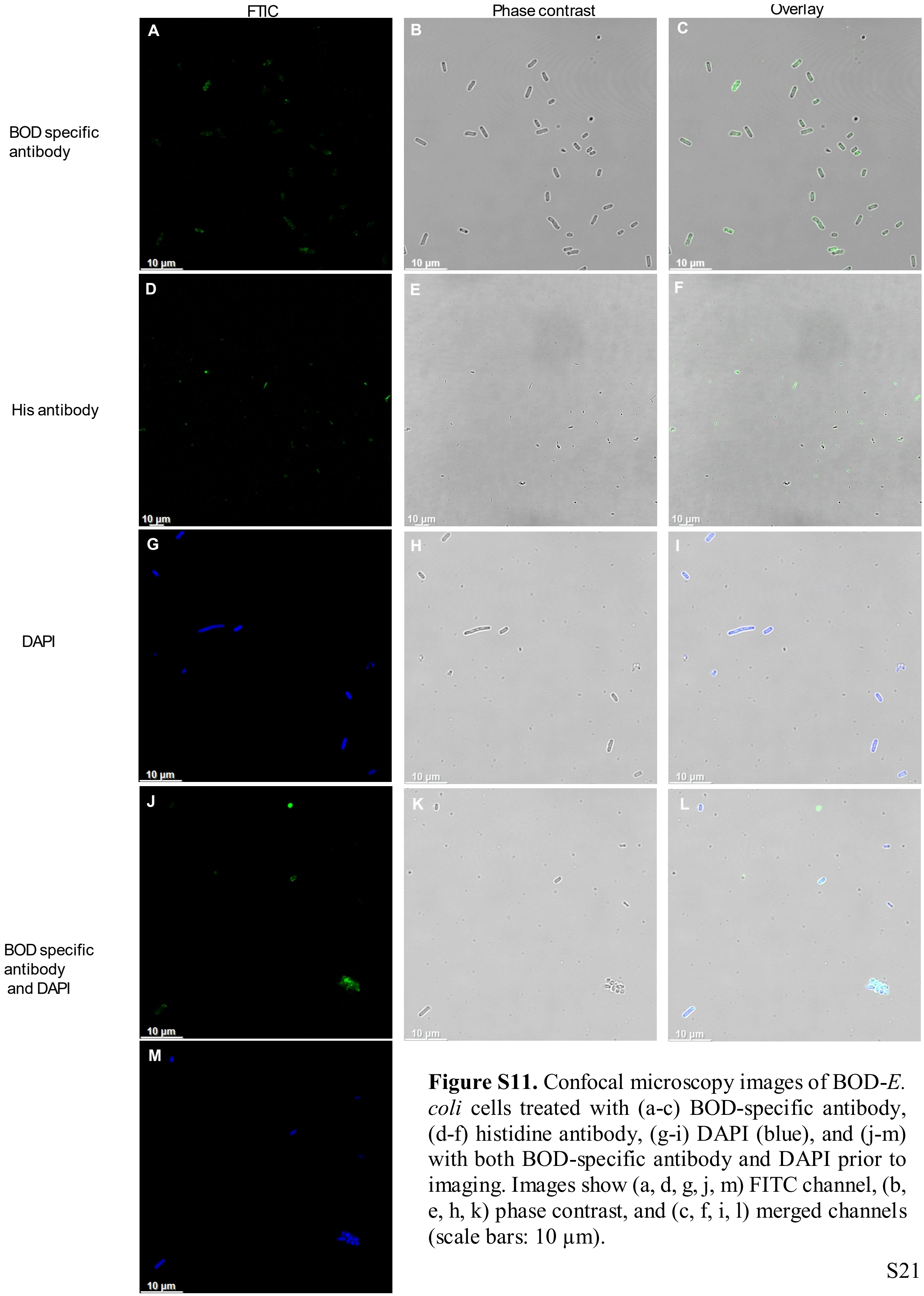

Figure S11. Confocal microscopy images of BOD-*E. coli* cells treated with (a-c) BOD-specific antibody, (d-f) histidine antibody, (g-i) DAPI (blue), and (j-m) with both BOD-specific antibody and DAPI prior to imaging. Images show (a, d, g, j, m) FITC channel, (b, e, h, k) phase contrast, and (c, f, i, l) merged channels (scale bars: 10 µm).

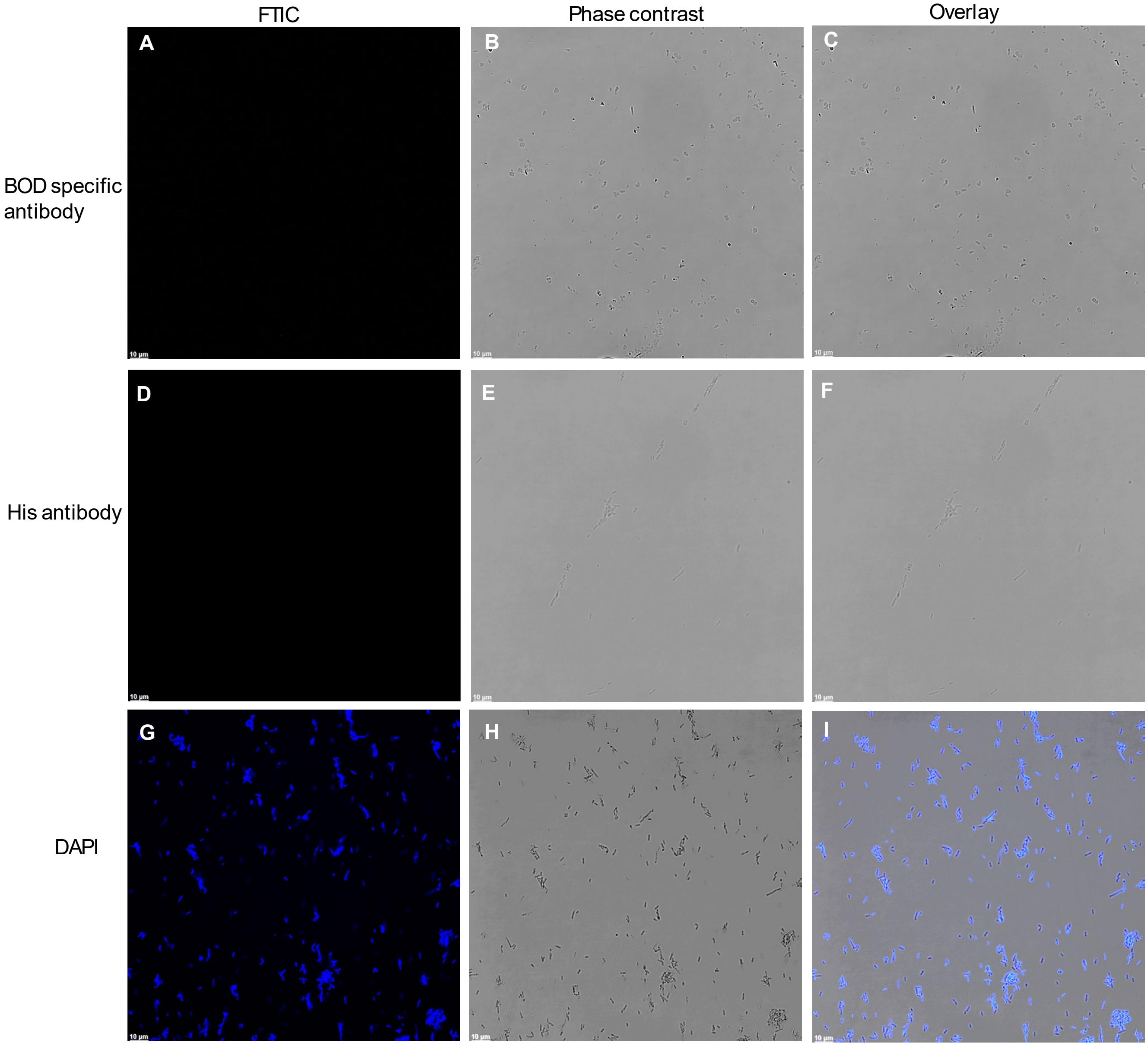

Figure S12. Confocal microscopy images of apoBOD-*E. coli* cells treated with (a-c) BOD-specific antibody, (d-f) histidine antibody, and (g-i) DAPI prior to imaging. Images show (a, d, g) FITC channel, (b, e, h) phase contrast, and (c, f, i) merged channels (scale bars: 10 µm).

7. Estimation of holo-BOD quantity immobilized on the electrode

The ICP-MS analysis indicates that 50% of BOD molecules on *E. coli* surface were effectively reconstituted. Based on that, it is estimated that there are 25,000-35,000 holo-BOD molecules per cell. As 50 μL of BOD-*E. coli* suspension (OD_{600} = 2.2) was dropped-casted on a 1 cm^2 carbon cloth electrode and the mass of one BOD molecule is 68 kDa (1.13 × 10^{-19} g), the mass of holo-BOD immobilized on the electrode was calculated as follows:

$$\frac{8\times10^{8}\ \text{cell/mL}}{1\ (\text{OD}_{600})}\times 2.2\ (\text{OD}_{600})\times 0.050\ mL = 8.8\times10^{7}\ \text{cell/electrode}$$

$$8.8\times10^{7}\frac{\text{cell}}{\text{electrode}}\times 35{,}000\frac{BOD}{cell}\times\frac{1.13\times10^{-19}\text{g}}{\text{BOD}} = 3.5\times10^{-4}\ \text{mg/electrode}$$

The calculation of TOF was based on the estimated number of holo-BOD on the electrode surface (3.08 × 10^{12} BOD molecules/electrode). The current of the cyclic voltammogram and of the current-time curve normalized by estimated holo-BOD mass on the carbon electrode are shown:

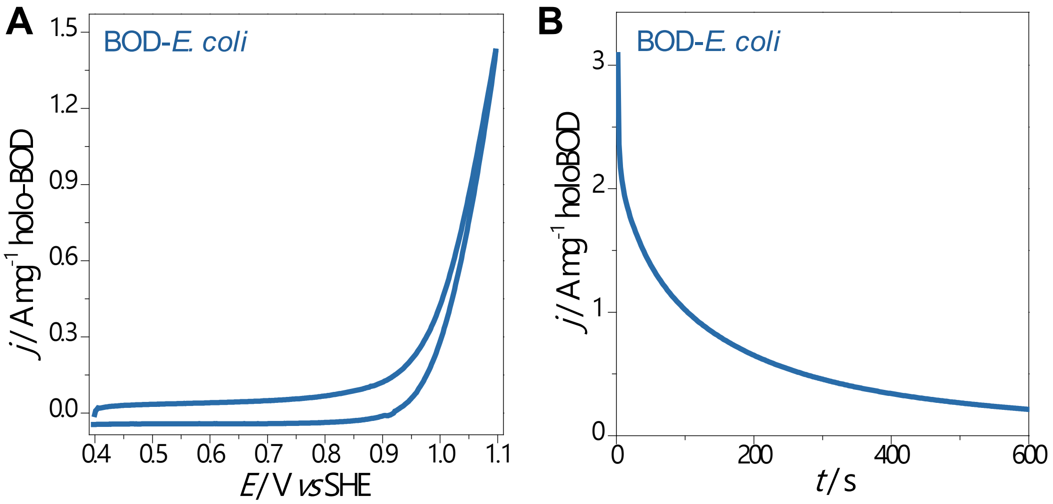

Figure S13. A) Cyclic voltammograms at 25 mV s^{-1} and (B) current-time curves at 1.10 V recorded with BOD-*E. coli*. Current values were normalized by the holo-BOD mass in the electrodes.

8. BOD-INP hydropathy analysis

The engineered fusion protein consists of 761 amino acids, comprising a 534-residue BOD domain and a 227-residue INPNC domain at the C-terminus. Within the INPNC segment, we identified a substantial enrichment of polar residues, particularly serine (S) and threonine (T), which together account for 36 residues—corresponding to approximately 16% of the domain. Notably, a highly ordered decapeptide motif, STSTSTSTST, is present in the central region of the INPNC domain (residues 112–121), forming a canonical repeat structure implicated in water structuring in ice-nucleating proteins. While this motif constitutes only ~4.5% of the total INPNC sequence, it represents a locally concentrated and spatially exposed hydration-enabling segment.

To further explore the hydration potential of this domain, we performed a Kyte–Doolittle hydropathy analysis[22] using a 21-residue sliding window (Figure S13). The BOD domain (residues 1–534) exhibited a relatively balanced hydropathy profile, with interspersed polar and mildly hydrophobic segments. In contrast, the INPNC domain (residues 534–760) displayed an overall heterogeneous pattern, including several strongly hydrophilic regions with hydropathy values approaching –1.5, alongside interspersed hydrophobic peaks. These alternating segments reflect the mosaic-like nature of the INP sequence, where localized polar motifs are embedded within a repetitive scaffold. While these results do not provide definitive evidence for the presence of water channels, they suggest the existence of localized polar regions that may aid water delivery toward the protein's active sites.

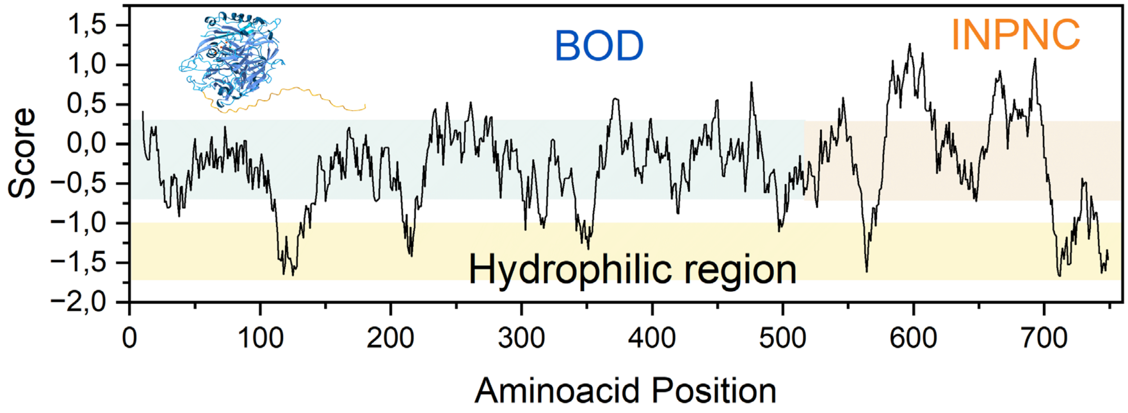

Figure S14. Kyte–Doolittle hydropathy profile of the 760-residue BOD-INPNC fusion protein, computed with a 21-residue sliding window. The BOD domain (residues 1–534) shows moderately hydrophobic regions interspersed with neutral and polar segments. In contrast, the C-terminal INPNC domain (residues 534–760) exhibits a pronounced shift toward negative hydropathy values, consistent with increased surface polarity and water affinity.

9. Electrochemical monitoring of O_2 generated by BOD-decorated cells

We employed our previously-developed assay developed to monitor OER.[23] Briefly, an amperometric sensor based on Pt-catalyzed ORR was used.[24,25] The O_2 reduction reaction occurs on a Pt wire polarized at -0.30 V vs SHE and is monitored by chronoamperometry at the wire. This reaction is coupled WOR performed by the BOD-*E. coli* on a carbon cloth electrode polarized to 1.1 V vs. SHE. The system was calibrated in the presence and absence of dissolved O_2 in the electrolyte solution used to run the oxygen sensing reaction (phosphate solution, pH 9.0). The response curve at the Pt wire is shown in Figure S15 for BOD-*E. coli* (blue) and uninduced cells (red). When the applied potential at the carbon electrode was interrupted (potential off, Figure S15), the O_2 reduction current significantly decreases.

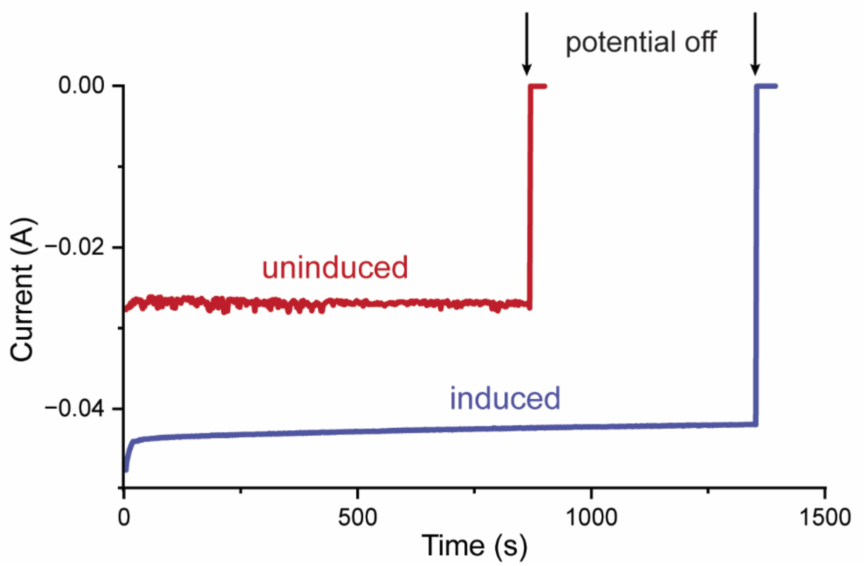

Figure S15. Chronoamperometry curves recorded at a Pt wire to detect dissolved O_2 based on performance of the ORR. Electrodes were 1 cm^2 carbon cloth modified with *E. coli*.

10. ROS detection

To confirm that O_2 is the primarily-produced product and that radicals generated from ORR are not present, we evaluated the electrolyte solution used to perform the WOR with a nitro blue tetrazolium chloride (NBT) assay. This assay detects reductive radicals such as superoxide by generating a formazan derivative of the NBT compound.[26] Briefly, NBT was dissolved in DMSO, and sample in phosphase solution (pH 9.0) was added in equal volume. UV-Vis measurements were made immediately following mixing of the solution (Figure S16). For the positive control (+H_2O_2), NaOH and H_2O_2 were added to generate superoxide *in situ* (green). The negative control (-H_2O_2) had NaOH added but no hydrogen peroxide (gray). Electrolyte from a carbon electrode modified with induced BOD-*E. coli* (blue) did not show any radical ORR products, neither did electrolyte from uninduced cells (red).

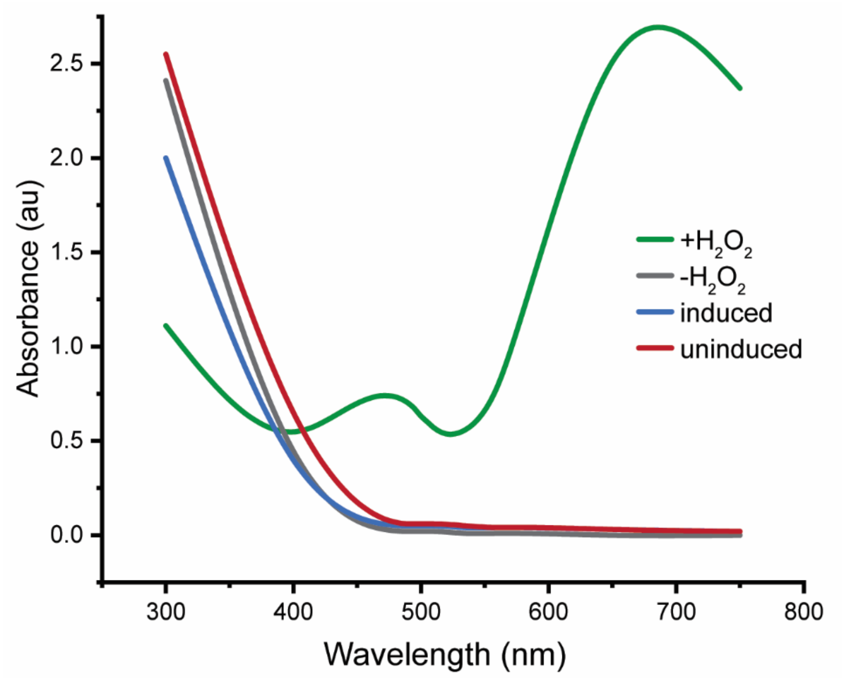

Figure S16. UV-Vis measurements of NBT assay for reductive oxygen radicals. Superoxide is generated *in situ* with NaOH and H_2O_2, which reacts with NBT to generate a formazan compound with a characteristic absorbance spectrum (green). In the absence of superoxide, the formazan is not generated (gray). Neither BOD-*E. coli* or uninduced *E. coli* generate reductive radicals such as superoxide.

11. Computational details

The molecular figures were generated using ChimeraX 1.8.[27] The lipid membrane model was constructed using CHARMM-GUI[28–31] and designed to mimic the surface composition of *Escherichia coli*. The protein structure of BOD, in combination with INP, was obtained from the AlphaFold Protein[32,33] Structure Database (UniProt entry AF-Q12737-F1-v4, https://alphafold.com/entry/Q12737). The AlphaFold-predicted structure has a mean pLDDT (predicted Local Distance Difference Test) score of 94.66, indicating high confidence in the majority of the protein. Variability in the pLDDT scores reflects differing levels of confidence, with regions such as flexible loops and termini predicted with lower confidence. While the AlphaFold model provides a reliable starting point, it should be noted that computationally predicted structures may not fully represent native conformations under all conditions. The visualization model integrates the AlphaFold protein structure with the lipid bilayer to illustrate the spatial arrangement of the system. This approach provides a detailed representation of the protein-membrane environment, enabling qualitative analyses and structural insights.

REFERENCES

[1] A. L. Furst, A. C. Hoepker, M. B. Francis, *ACS Cent. Sci.* **2017**, *3*, 110.

[2] P. S. Daugherty, M. J. Olsen, B. L. Iverson, G. Georgiou, *Protein Eng.* **1999**, *12*, 613.

[3] P. S. Daugherty, *Curr. Opin. Struct. Biol.* **2007**, *17*, 474.

[4] J. Rockberg, J. Löfblom, B. Hjelm, M. Uhlén, S. Ståhl, *Nat. Methods* **2008**, *5*, 1039.

[5] R. Zhang, N. Ye, Z. Wang, S. Yang, J. Li, *Cell. Mol. Bioeng.* **2024**, *17*, 453.

[6] Y. Gotoh, Y. Kondo, H. Kaji, A. Takeda, T. Samejima, *J. Biochem.* **1989**, *106*, 621.

[7] S. Alon, K. K. Dan, A. Lital, *Chem. Commun.* **2012**, *48*, 49.

[8] Y. Chen, B. Stemple, M. Kumar, N. Wei, *Environ. Sci. Technol.* **2016**, *50*, 8799.

[9] A. Paul, T. A. R. Silva, M. M. A. Soliman, J. Karačić, B. Šljukić, E. C. B. A. Alegria, R. A. Khan, M. F. C. Guedes da Silva, A. J. L. Pombeiro, *Int. J. Hydrogen Energy* **2022**, *47*, 23175.

[10] Y. Liu, Z. Wei, Z. Li, S. Wu, S. Qiao, H. Zhou, *Int. J. Hydrogen Energy* **2024**, *61*, 986.

[11] C. Gautam, A. Singh, A. Singh, A. K. Singh, V. K. Sharma, P. Kumar, *J. Mol. Struct.* **2021**, *1243*, 130928.

[12] Q. Wang, Y. Du, Y. Gong, W. Xiao, H. Li, Y. Du, G. Xu, Z. Wu, L. Wang, *Chem. Eng. J.* **2024**, *489*, 151322.

[13] P. Mohana, R. Yuvakkumar, G. Ravi, S. Arunmetha, *Electrochim. Acta* **2024**, *473*, 143464.

[14] G. C. Sedenho, S. Q. Nascimento, M. Zamani, F. N. Crespilho, A. L. Furst, *Adv. Sci.* **2024**, *11*, 1.

[15] A. M. Geer, C. Musgrave, C. Webber, R. J. Nielsen, B. A. McKeown, C. Liu, P. P. M. Schleker, P. Jakes, X. Jia, D. A. Dickie, J. Granwehr, S. Zhang, C. W. Machan, W. A. Goddard, T. B. Gunnoe, *ACS Catal.* **2021**, *11*, 7223.

[16] H. A. Younus, Y. Zhang, M. Vandichel, N. Ahmad, K. Laasonen, F. Verpoort, C. Zhang, S. Zhang, *ChemSusChem* **2020**, *13*, 5088.

[17] S. Khan, S. Sengupta, M. A. Khan, M. P. Sk, N. C. Jana, S. Naskar, *Inorg. Chem.* **2024**, *63*, 1888.

[18] P. Ye, K. Fang, H. Wang, Y. Wang, H. Huang, C. Mo, J. Ning, Y. Hu, *Nat. Commun.* **2024**, *15*, 1012.

[19] R. Zhang, Q. Wu, Y. Han, Y. Zhang, X. Wu, J. Zeng, K. Huang, A. Du, J. Chen, D. Zhou, X. Yao, *Small* **2025**, *21*, 2408266.

[20] B. Yao, Y. Chen, Y. Yan, Y. Yang, H. Xing, Y. Xu, D. Jiao, Z. Xing, D. Wang, X. Yang, *Angew. Chemie - Int. Ed.* **2025**, *64*, e202416141.

[21] X. Yang, W. Wu, X. Chen, F. Wu, S. Fan, P. Yu, L. Mao, *Sci. Adv.* **2022**, *8*, eabo3315.

[22] J. Kyte, R. F. Doolittle, *J. Mol. Biol.* **1982**, *157*, 105.

[23] G. Sedenho, S. Q. Nascimento, M. Zamani, F. N. Crespilho, A. L. Furst, *Adv. Sci.* **2024**, *11*, 2402234.

[24] N. J. Finnerty, F. B. Bolger, *Bioelectrochemistry* **2018**, *119*, 124–135.

[25] L. Rivas, S. Dulay, S. Miserere, L. Pla, S. B. Marin, J. Parra, E. Eixarch, E. Gratacós, M. Illa, M. Mir, J. Samitier, *Biosens. Bioelectron.* **2020**, *153*, 112028.

[26] H. S. Choi, J. W. Kim, Y.-N. Cha, C. Kim, *J. Immunoassay Immunochem.* **2006**, *27*, 31-44.

[27] E. C. Meng, T. D. Goddard, E. F. Pettersen, G. S. Couch, Z. J. Pearson, J. H. Morris, T. E. Ferrin, *Protein Sci.* **2023**, *32*, e4792.

[28] S. Jo, T. Kim, V. G. Iyer, W. Im, *J. Comput. Chem.* **2008**, *29*, 1859.

[29] E. L. Wu, X. Cheng, S. Jo, H. Rui, K. C. Song, E. M. Dávila-Contreras, J. L. Yifei Qi, V. Monje-Galvan, R. M. Venable, J. B. Klauda, W. Im, *J. Comput. Chem.* **2014**, *35*, 1997.

[30] S. Jo, J. B. Lim, J. B. Klauda, W. Im, *Biophys. J.* **2009**, *97*, p50.

[31] S. Feng, S. Park, Y. K. Choi, W. Im, *J. Chem. Theory Comput.* **2023**, *18*, 2161.

[32] J. Jumper, R. Evans, A. Pritzel, T. Green, M. Figurnov, O. Ronneberger, K. Tunyasuvunakool, R. Bates, A. Žídek, A. Potapenko, A. Bridgland, C. Meyer, S. A. A. Kohl, A. J. Ballard, A. Cowie, B. Romera-Paredes, S. Nikolov, R. Jain, J. Adler, T. Back, S. Petersen, D. Reiman, E. Clancy, M. Zielinski, M. Steinegger, M. Pacholska, T. Berghammer, S. Bodenstein, D. Silver, O. Vinyals, A. W. Senior, K. Kavukcuoglu, P. Kohli, D. Hassabis, *Nature* **2021**, *596*, 583.

[33] M. Varadi, D. Bertoni, P. Magana, U. Paramval, I. Pidruchna, M. Radhakrishnan, M. Tsenkov, S. Nair, M. Mirdita, J. Yeo, O. Kovalevskiy, K. Tuny, A. Žídek, H. Tomlinson, D. Hariharan, J. Abrahamson, T. Green, J. Jumper, D. Hassabis, S. Velankar, *Nucleic Acids Res.* **2024**, *52*, D368.
